# Detecting complex infections in trypanosomatids using whole genome sequencing

**DOI:** 10.1186/s12864-024-10862-6

**Published:** 2024-10-29

**Authors:** João Luís Reis-Cunha, Daniel Charlton Jeffares

**Affiliations:** York Biomedical Research Institute, Department of Biology and York Biomedical Research Institute, University of York, York, YO10 5DD UK

**Keywords:** Complex infections, Polyploidy, Multiplicity of infection, Trypanosomatids, Aneuploidy, Protozoan parasites

## Abstract

**Background:**

Trypanosomatid parasites are a group of protozoans that cause devastating diseases that disproportionately affect developing countries. These protozoans have developed several mechanisms for adaptation to survive in the mammalian host, such as extensive expansion of multigene families enrolled in host-parasite interaction, adaptation to invade and modulate host cells, and the presence of aneuploidy and polyploidy. Two mechanisms might result in “complex” isolates, with more than two haplotypes being present in a single sample: multiplicity of infections (MOI) and polyploidy. We have developed and validated a methodology to identify multiclonal infections and polyploidy using whole genome sequencing reads, based on fluctuations in allelic read depth in heterozygous positions, which can be easily implemented in experiments sequencing genomes from one sample to larger population surveys.

**Results:**

The methodology estimates the complexity index (CI) of an isolate, and compares real samples with simulated clonal infections at individual and populational level, excluding regions with somy and gene copy number variation. It was primarily validated with simulated MOI and known polyploid isolates respectively from *Leishmania* and *Trypanosoma cruzi*. Then, the approach was used to assess the complexity of infection using genome wide SNP data from 497 trypanosomatid samples from four clades, *L. donovani/L. infantum*, *L. braziliensis*, *T. cruzi* and *T. brucei* providing an overview of multiclonal infection and polyploidy in these cultured parasites. We show that our method robustly detects complex infections in samples with at least 25x coverage, 100 heterozygous SNPs and where 5–10% of the reads correspond to the secondary clone. We find that relatively small proportions (≤ 7%) of cultured trypanosomatid isolates are complex.

**Conclusions:**

The method can accurately identify polyploid isolates, and can identify multiclonal infections in scenarios with sufficient genome read coverage. We pack our method in a single R script that requires only a standard variant call format (VCF) file to run (https://github.com/jaumlrc/Complex-Infections). Our analyses indicate that multiclonality and polyploidy do occur in all clades, but not very frequently in cultured trypanosomatids. We caution that our estimates are lower bounds due to the limitations of current laboratory and bioinformatic methods.

**Supplementary Information:**

The online version contains supplementary material available at 10.1186/s12864-024-10862-6.

## Background

Trypanosomatid parasites are a group of protozoans that cause devastating diseases, imposing severe health and economic burdens primarily upon developing countries [1–3]; (https://www.paho.org/en/topics/chagas-disease*).* Among them, African trypanosomiasis, American trypanosomiasis and leishmaniasis, caused respectively by *Trypanosoma brucei*; *Trypanosoma cruzi* and species from the *Leishmania* genus are Neglected Tropical diseases (NTDs), with more than one billion people living at risk of infection. These diseases are a part of the WHO NTDs elimination road map for 2021–2030 (WHO/UCN/NTD/2020.01) [3].

Various mechanisms for immune evasion and adaptation to survive in the mammalian host have evolved in these parasites; such as antigenic variation in the extracellular parasite *T. brucei* [4–7]; extensive expansion of multigene families enrolled in host-parasite interaction in *T. cruzi* [8–10]; adaptation to invade and modulate host cells in *T. cruzi* and *Leishmania* [11–13]; and the presence of aneuploidy and polyploidy [14–16]. Genome instability, observable within population by variation in chromosome copy numbers [14], and frequent formation of triploids and tetraploids [17–20] are also features of these species. There is also evidence of the occurrence of multiplicity of infections (MOI) both in the mammalian and in the insect vector, where more than one diploid parasite genotype is observed in the same host. MOI might have consequences to the parasite biology [21–28] and is important for the resulting meiotic recombination within the vectors [29]. Both MOI and allopolyploidy will result in complex isolates, with more than two haplotypes being present in a single sample.

The complexity of natural infections is relevant to understanding trypanosomatid biology and disease control, as MOI cases provide direct evidence for genetically diverse infections that could increase the speed in which virulence and drug resistance genes may be shared in the population. In general, parasite diversity allows sub-populations to be selected in different environments, increasing adaptability [21, 28, 30].

MOI has already been described in *Leishmania* infections [22, 23, 31], where there is usually a dominant genotype combined with rare genotypes of the *same* species [32], and *different* species of the parasite may cohabit the same host [33]. This can result in interspecies hybrids when it occurs in the insect vector [34]. Multiclonal infections were also described in *T. cruzi* using microsatellite and marker genes, where it appears to be more prevalent in mammalian reservoirs, such as rodents and opossums, when compared to human patients [24–26]. There is also evidence of MOI in *T. brucei* in the mammalian host [27], and in the inset vector [35]. Coinfection with two strains in the mammalian host leads to competitive suppression in *T. brucei*, enhancing host survival [36], and also impact clinical outcomes in *T. cruzi* [37] and *Leishmania* [38]; reinforcing that MOI may impact patient clinical outcomes in these parasites.

Hybridization leading to temporary trisomy/tetraploidy was already demonstrated in trypanosomatids. In *T. cruzi*, experimental hybrids originated from diploid parental strains were mostly tetraploid, and underwent genome erosion throughout culture passages, reverting to trisomy [17]. In *Leishmania*, hybridization was shown to generate diploid, triploid or tetraploid strains [39], both in intra species [40, 41], as well as between species hybrids [42]. This transient presence of four haplotypes (in allotetraploids) in a single cell might increase genetic exchange and recombination, increasing the potential variability, as the parasites revert back to trisomy and disomy by genome erosion.

Several methods have already been proposed to estimate haplotype phasing, hybridization and multiclonal infections, such as STRUCTURE [43], Beagle [44], Admixture [45] and PoolHap [46]. Several of these methods require population-wide data to identify haplotypes or populations; or might be heavily impacted by repetitive region variation that are common in trypanosomatids. In the present work, we have developed an alternative and complementary methodology to identify multiclonal infections and polyploidy in diploid species using whole genome sequencing (WGS) reads, based on fluctuations in allelic read depth in heterozygous positions. This methodology uses the complexity index (CI) proposed in Franssen et al. [31]. We parameterize this metric by comparing the allelic read depth at heterozygous sites in real samples to simulated clonal infections, which were generated using allelic read depths sampling by binomial trials to generate stochastic allelic depths. This approach was used to assess the complexity of infection in 497 trypanosomatid samples, a combination of primary isolate/stock, clones and strains from four species/complexes, *L. donovani/L. Infantum* (*L. donovani* complex), *L. braziliensis*, *T. cruzi* and *T. brucei* based on genome-wide markers, providing an overview of multiclonal infection and polyploidy in these parasites. We show that our method robustly detects complex infections with at least **25x** coverage and at least 100 heterozygous SNPs. We find that a relatively small proportion (≤ 7%) of cultured trypanosomatid isolates are complex. For methodological reasons, these proportions represent a lower bound of complex infections in these species.

## Methods

### Overview

We define the complexity index (CI) as the deviation from the expected 50% of reads in each allele in heterozygous positions, as proposed in Franssen 2021 [31]. It is estimated by the absolute value of the difference between the alternate allele read depth (AARD) in heterozygous positions and 0.5, the expected AARD in diploid, clonal heterozygous SNPs.

**Fig. 1:**
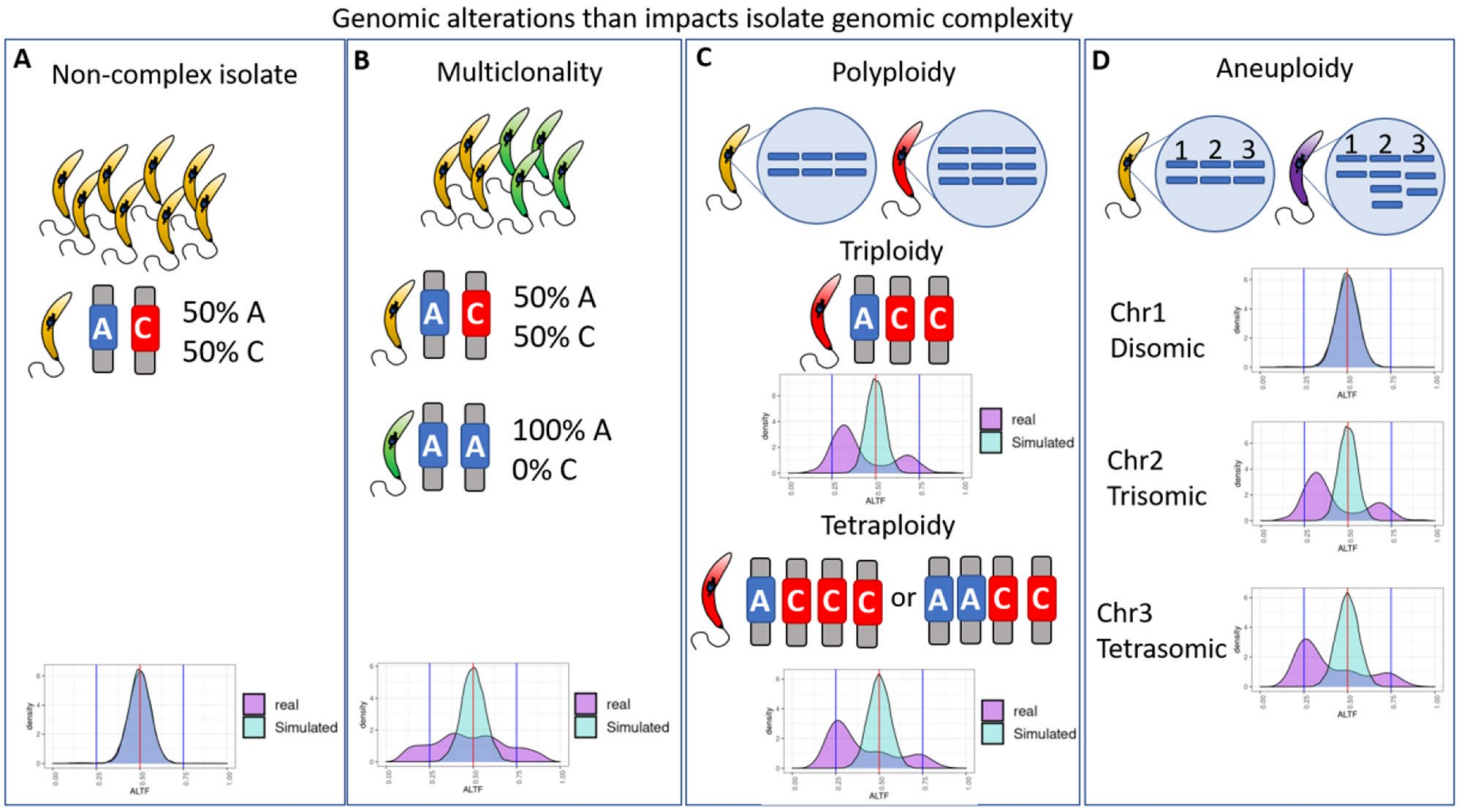
Phenomena that may alter the complexity of trypanosomatid isolates. (**A**) Non-complex clonal, euploid, diploid isolate. In this example, the proportion of AARD in a heterozygous position is expected to be close to 0.5, and the genomic distribution of AARD in the real sample (purple) is similar to the simulated-clonal isolate generated from random-draw binomial trials (cyan). (**B**) Multiclonality: having more than one parasite clone in an isolate, will result in deviations from the expected distribution and mean of 0.5 AARD, as the different clones may have different SNP sites or a SNP position may be homozygous in one clone and heterozygous in the other. This will result in different distributions of genome-wide AARD and a higher CI, which also vary depending on the proportion of the secondary clone (see Fig. 2). (**C**) Polyploidy, having extra copies of the whole chromosomal set also alters the AARD distribution. While triploid isolates have AARD peaks of ~ 0.33 and ~ 0.66, tetraploid isolates will have a combination of AARD peaks in ~ 0.25, ~ 0.5 and ~ 0.75. Higher ploidy would result in even more complex patterns (**D**) Aneuploidy, having an unbalanced number of chromosomal copies will result in different AARD distributions in different chromosomes, which could impact whole genome CI estimations

The CI can be impacted by several phenomena, including multiclonality (multiple clones or genotypes in a sample, often present in different proportions), polyploidy (multiple copies of all chromosomes) and aneuploidy (multiple copies of some chromosomes). (Fig. 1). In a non-complex clonal, euploid, diploid isolate, the mean AARD, meaning the proportion of reads that correspond to the alternate allele in each heterozygous position, is expected to be close to 0.5, as there will be a similar number of reads mapping in both alleles (Fig. 1A). Hence, when all heterozygous SNPs in a genome are evaluated, the distribution of the AARDs will have a peak at 0.5. Some phenomena that have already been observed in trypanosomatids, such as multiclonality (Fig. 1-B), polyploidy (Fig. 1-C) and aneuploidy (Fig. 1-D) will alter this proportion, changing the distribution peaks or flattening their curve, which may be seen in density plots of AARD values for all heterozygous SNPs in a sample.

To provide a numeric and statistical large-scale evaluation of this deviation from expected AARD, we estimated CI: the absolute value of the deviation from the expected 0.5 proportion in each heterozygous SNP position; and compared real samples with simulated clonal isolates at individual and populational level. We have estimated cutoffs for complex isolates based on the mean complexity of simulated clonal isolates from population genomic data from various trypanosomatid clades, and used the Cochran-Mantel-Haenszel (CMH) test to support the evaluation in each isolate.

### Heterozygous SNP calling and alternate allele read depth (AARD) estimation

Representative whole genome sequencing (WGS) read data from trypanosomatid isolates were downloaded from the National Centre for Biotechnology Information (NCBI) Sequence Read Archive (SRA) using Fastq-dump [47]. Only Illumina sequencing reads from publicly available datasets were used (Supplementary Table 1, Additional File 1; Supplementary Table 2, Additional File 2; Supplementary Table 3, Additional File 3; Supplementary Table 4, Additional File 4; Supplementary Table 5, Additional File 5; Supplementary Table 6, Additional File 6; and Supplementary Table 7, Additional File 7). Each read library was filtered using fastp v2.10.7 [48], with the parameters: average Q20, minimal length 50 and removing the read extremities with base quality lower than Q25. Next, for each species, the reads were mapped to an appropriate reference genome, listed in Supplementary Table 8, Additional File 8 using BWA-mem v.0.7.17 [49], retaining only reads with mapping quality 30 or higher and removing PCR duplicates using SAMtools v.1.10 [50]. The number of mapped reads was estimated using SAMtools v.1.10. The genome coverage was estimated by the mean coverage of all single copy genes in the genome, using SAMtools depth. The single copy genes were selected using OrthoFinder v. 2.5.4 [51].

For the SNP calls, read groups were assigned for the filtered mapped read libraries, using PicardTools v.2.21.6 (https://github.com/broadinstitute/picard). SNPs and indels were called using the Genome Analysis Toolkit (GATK) v.4.1.0.0 HaplotypeCaller and Freebayes v. 1.3.5 (https://github.com/ekg/freebayes), with a minimum alternative allele read count of 5. Only SNP/Indel positions that were identified by both callers were kept. For each dataset, the single-sample VCFs were merged with VCFtools v.0.1.16 and regenotyped using Freebayes. Next, the VCF file was filtered using BCFtools v.1.12 [52], to select only biallelic SNPs, with call quality above 200, coverage greater than half of the mean genome coverage (i.e, at least haploid), and lower than twice the genome coverage (i.e. is not duplicated) with mapping quality 40 or higher and properly paired reads (-m2 -M2 -i' TYPE="snp” & QUAL > 200 & INFO/DP > Cov/2 & INFO/DP < Cov*2 & INFO/MQM > 40 & INFO/MQMR > 40 & INFO/PAIRED > 0.9 & INFO/PAIREDR > 0.9). The only exception was the *T. cruzi* dataset, as several samples were single-end reads, so the “INFO/PAIRED > 0.9 & INFO/PAIREDR > 0.9” were not used. To remove SNP call bias from repetitive regions and paralogous genes, only SNPs in single copy genes were used in subsequent analysis. After filtering, the multisample VCF was split into single sample VCFs, to be used in the complexity pipeline (see below). For the individual sample VCFs, only SNP positions with read depth ≥ 5 in both the reference and alternate alleles were considered as heterozygous. SNPs where the read depth in one allele was > 5, and between 1 and 4 in the other allele were classified as dubious, and not used in the complexity estimation. This was a conservative measure to remove potential noise and sequencing/mapping errors.

To control the bias of aneuploidy in the CI estimation, chromosome(s) with coverage higher than 1.15x or lower than 0.85x of the genome coverage in a sample were excluded from downstream analysis. Similarly, to mitigate bias from gene copy number variants (CNVs), SNPs in genes with coverage higher than 1.15x or lower than 0.85x of its chromosome coverage were also removed. The gene coverage was estimated using SAMtools depth and the gene coordinates from the General Feature Format (GFF) obtained in TriTrypDB v.55. The chromosomal somy for each sample was estimated using the median read depth coverage of single copy genes in each chromosome with non-outlier coverage (Grubb’s tests, with *P* < 0·05), normalised by genome coverage. Data from *Leishmania* and *Trypanosoma cruzi* chromosomes 31 were always excluded, as they are consistently supernumerary in all isolates from these species [14]. Only read libraries with genome coverage ≥ 25x were used in posterior analysis.

### Complexity evaluation, Cochran-Mantel-Haenszel (CMH) estimation and AARD distribution

The classification of an isolate as complex was based on comparisons between the real data with simulated clonal isolates. Samples that were classified as complex had to have: A higher CI than clonal simulated isolates, a significant CMH p-value associating the real sample to deviations from the expected allele read counts, and an AARD distribution that deviates from the simulated clonal isolate. Only samples that were above both Complexity and CMH cutoffs were assumed to be complex. Details are described below.

#### Complexity

For each SNP site *i, CIi* is the absolute value of the difference between the AARD in that position and the expected AARD in diploid, non-mixed SNP positions, within a sample (which is expected to be close to 0.5). To account for the random sampling of reads sequenced from each allele of heterozygous sites, a simulated “clonal-diploid” SNP data sample was generated for each isolate in each population, with the same number of SNPs and read depth as in the real sample, using series of binomial trials. For each SNP position (*i*) in the real sample, we conducted *n* binomial trials, by randomly sampling from a binary array (0 or 1), where 0 represents the reference allele and 1 the alternate allele, where *n* is the read depth in the position in the real sample. The *AARDi* for the *i*th position in the simulated clone was the sum of the binomial trials (*b*), divided by the total read coverage at site *i* (*n*);

$$\:AARDi\:=\:\frac{{\sum\:}_{1}^{n}b\:}{n}$$

and the complexity index of this position (*CI*_*i*_) was calculated as the absolute difference between the expected AARD of 0.5

$$\:CIi\:=\:\:\left|AARDi\:-\:0.5\right|\:$$

The CI of the isolate with *i* heterozygous SNPs is calculated as the mean of all *CIi* values. To classify an isolate as “potentially complex” the CI had to be higher than the mean + 3 standard deviations (SD) from all simulated clonal isolates in the population. For an isolate to be classified as “complex” it had to have a CI value > 0.1, which is slightly higher than the cutoff for the simulated data for all trypanosomatid populations (see results section). We recommend the CI threshold of 0.1 be used to classify samples in projects with a small number of samples.

#### CMH test

Another metric used to assess the isolate complexity was the CMH test, which tests the association between binary predictors (expected counts of reference and alternate alleles to generate the expected AARD of 0.5) and binary outcomes (observed counts of reference and alternate alleles) considering stratification from a third variable (in our case the position in the genome). In this case, it was used to compare the combined effect of all SNP read depth in each allele to classify an isolate as complex, comparing real samples and binomial trial simulated clonal-diploid samples. For each isolate with *i* heterozygous SNP positions, CMH p-values were generated using *i* 2 × 2 contingency tables using row 1; actual read depth of each allele at position *i*, row 2; the result of *n* binomial trials (where *n* is the total read depth at site *i*), and columns being the reference and alternate allele counts. We found that a significance level of *p* ≤ 10^− 10^ was a reasonable CMH test threshold Supplementary Fig. 1, Additional File 9. R scripts for both tests are available on GitHub (https://github.com/jaumlrc/Complex-Infections.git).

#### AARD distribution

The AARD distribution for all SNP positions in the real sample and its paired simulated clonal sample were generated in R, and deviations from their distributions were accounted as evidence for complexity.

### Assessing complexity on mixed samples

To estimate the accuracy of the combined CI and CMH tests to identify multiclonal infections with different proportions of the secondary clone, a collection of 24 *L. donovani* clones from East Africa (EA) described in Zackay 2018 [53] were used (Supplementary Table 1, Additional File 1). To create artificial multiclonal samples in silico and assess the impact of the proportion of a secondary clone in complexity estimations, three clones, ERR205809, ERR205816 and ERR205819, were selected. These clones were selected as they are from the same species (*L. donovani*) and originated from different primary isolate/stock, respectively GR356, GR383 and GR364 [53]. Each of these three read libraries was combined with the full library of another of these three clones, where the secondary strain was downsampled to 2.5, 5, 10, 15, 25 and 50% of the full combined data, using SAMtools v.1.9 [50], to create ‘MIX’ samples. In each case, the main and secondary clones were permuted, generating a total of 33 combinations. The results from these artificially multiclonal samples were compared with those obtained from clones and simulated clonal isolates, based on their complexity index and CHM test, as previously described.

The impact of the number of heterozygous SNPs in the CI evaluation was examined by sub-sampling the number of SNP calls at random to 10, 50, 100, 300 SNPs, in 100 iterations, assessing the number of true positives (number of MIX samples that were classified as complex) and comparing with the estimations with the full set of SNPs.

Next, we evaluated the complexity of in silico mixed samples from 10 *T. cruzi* clones or strains (tcMIX), including two pairs of clones that originated from the same primary isolate: pair 1: SRR9643478 and SRR9643443 (clones from THY primary isolate/stock); and pair2: SRR9643494 and SRR964349 (clones from the TBM primary isolate/stock) (Supplementary Table 2, Additional File 2). The tcMIX samples were generated with a pairwise combination of each sample, with 5%, 10% and 20% of the secondary clone, as described for the *Leishmania* samples. This resulted in 86 expected complex tcMIX (10 clones x 9 other clones − 4 combinations of clones from the same primary isolate) for each proportion of the secondary clone; 10 clones, 30 self-samples (10 for each proportion of the secondary clone) and 12 tcMIX with “same isolate clones”, four for each proportion of the secondary clone: SRR9643478 (primary) and SRR9643443 (secondary), SRR9643443 (primary) and SRR9643478 (secondary), SRR9643494 (primary) and SRR9643495 (secondary) and SRR9643495 (primary) and SRR9643494 (secondary). The complexity results from the tcMIX were compared with those obtained from clones, combination of clones from the same primary isolate and simulated clonal isolates, based on their complexity index and CHM test, as previously described.

### Assessing complexity on polyploid samples

Besides multiclonality, another source of complexity is polyploidy, having extra full sets of chromosomal copies. To evaluate the impact of polyploidy in the CI estimations, we used samples from the *T. cruzi* dataset described in Matos 2022 [17], containing 8 diploid parental clones, and 11 triploid or tetraploid hybrids clones, where the somy of some samples were validated by flow cytometry [17] (Supplementary Table 3, Additional File 3). As performed for the multiclonal isolates, the SNPs counts for each sample were downsampled to 10, 50, 100, 300 or full set, in 100 iterations, and the accuracy to classify each group as complex was evaluated.

To further validate the method, we have also evaluated the complexity of *Leishmania* hybrids clones with known ploidies, described in Louradour 2021 [54] (Supplementary Table 4, Additional File 4) and in Cata-Preta 2022 [55] (Supplementary Table 5, Additional File 5).

### Assessing complexity on publically available trypanosomatids primary isolate/stocks, clones and strains

After validating our complexity estimations with simulated/controlled data, we went on to evaluate 497 WGS data sets from publicly available primary isolate/Stock and strains from four trypanosomatid species/complexes: *L. donovani/L. Infantum* (*L. donovani* complex), *L. braziliensis*, *T. cruzi* and *T. brucei* (Table 1, Supplementary Table 6, Additional File 6) [31, 54, 56–72]. We have also individually evaluated the interspecies hybrids from Sri Lanka, described in Lypaczewski 2021 (Supplementary Table 7, Additional File 7) [34]. Only read libraries from samples with read coverage ≥ 25x and where at least 100 heterozygous SNPs were called where used in this analysis. For these samples, the whole genome CI, as well as the CI for each chromosome was estimated.

The proportion of chromosomes that were used in the CI estimation was calculated for each isolate by dividing the number of chromosomes that were used in CI (had at least one identifiable SNP and were not aneuploid) by the total number of chromosomes in the species: *L. donovani* = 36; *L. braziliensis* = 35; *T. brucei* = 11; *T. cruzi* = 41. The proportion of complex chromosomes in an isolate was estimated dividing the number of chromosomes with complexity index ≥ 0.1 by the number of evaluated chromosomes. Even though we removed aneuploid chromosomes that had deviations from the mean genome coverage from each isolate, the intra isolate chromosome mosaicism (mosaic aneuploidy, and chromosome imbalance) [71, 73–76] may add noise to complexity measurements in field isolates, by having unbalanced values in a few chromosomes. Hence, we are only considering as “complex”, samples that had at least 50% of its evaluated chromosomes with a mean complexity value higher than 0.1.

The evaluation of statistical differences in the genome coverage and heterozygous SNPs/Kb in complex and non-complex samples was performed with Mann–Whitney U test, in R. The Pearson correlation between the genome coverage, SNPs/Kb and complexity was estimated in R. For both analyses the heterozygous SNPs/Kb were estimated dividing the heterozygous SNP numbers by the sum of the lengths of the single copy genes, in each set.

We also estimated the number of heterozygous SNPs in the maxicircle (kDNA) genome, as well as its coverage (as described for the nuclear genome). To identify kDNA SNPs, the maxicircle sequence (*L. donovani* = BK010877.1, *L. braziliensis* = OY748431, *T. cruzi* = MW732647, and *T. brucei* M94286.1; downloaded from NCBI) was combined with the genome reference for the read mapping. Only heterozygous SNPs that were outside the repetitive region and had at least 5% of the kDNA genome coverage were considered.

## Results

### Assessing the accuracy of the CI to identify multi-clonal and polyploid isolates, using simulated or controlled data

**Fig. 2:**
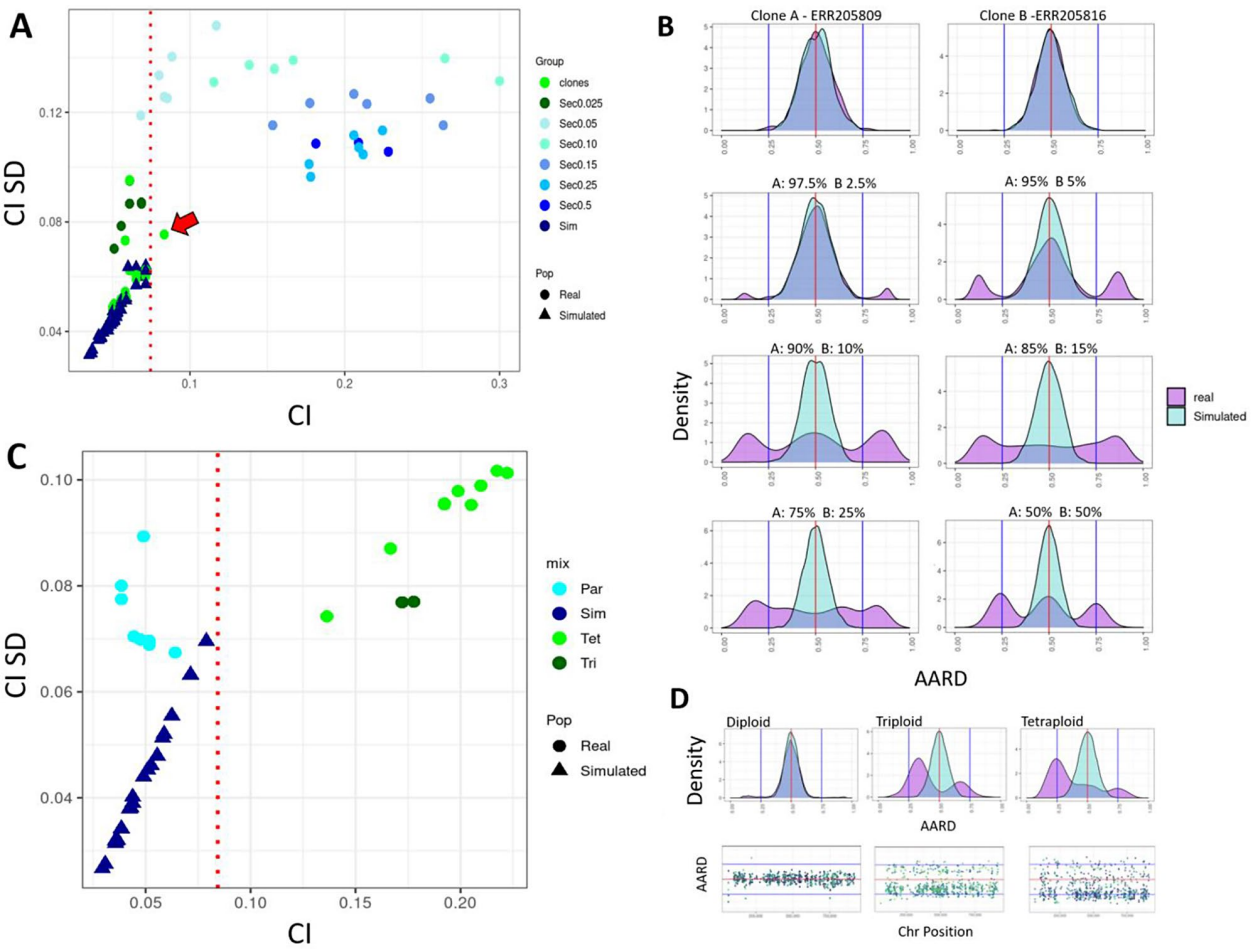
Assessing the impact of the proportion of the secondary clone and polyploidy in complexity estimates. (**A**) Complexity estimations in MIX samples. The X and Y axis represents, respectively, the mean CI and CI standard deviation. The sample origin is represented by shape, where each individual triangle represents a simulated clone, and circles represent the MIX samples. The proportion of the secondary clone is represented by colours. The red dotted line represents the complexity CI cutoff. The red arrow points to a clone that had a higher CI than the cutoff. However, its classification as complex was not supported by the CMH test. (**B**) Density distributions of the AARD proportion in heterozygous positions for increasing proportions of the secondary clone, varying from 2.5–50%. Each panel corresponds to a different isolate, including the clones and the MIX samples from ERR205809 (main) and ERR205816 (secondary). The purple distribution corresponds to the sample data, while the cyan distribution represents the simulated clone, with the same number of SNPs and read depth as the real sample. (**C**) Complexity estimations in each sample from the *T. cruzi* parental isolates and hybrid polyploid progeny. The X and Y axis represents, respectively, the CI and CI standard deviation. Each dot corresponds to an isolate (circles) or simulated clone (triangles), where parental diploid, triploid or tetraploid isolates are respectively represented in light-blue, dark-green and light green. Simulated clonal isolates are represented in dark-blue. The red dotted line represent the complexity CI cutoff. (**D**) Examples of diploid (SRR15686198), triploid (SRR15686206) and tetraploid (SRR15686203) AARD distributions plots. The top panels are density plots, where the purple distribution corresponds to the real data, while the cyan distribution represents the simulated clone, with the same number of SNPs and read depth as the real sample. Bottom panels represent the AARD distribution in each heterozygous SNP in the chromosome 36 from a diploid (SRR15686198), triploid (SRR15686206) and tetraploid (SRR15686203) sample. The Y axis represents the AARD and the X axis the chromosomal position

To evaluate the accuracy of the CI metric to identify multiclonal isolates, we created sequencing read data sets to represent multiclonal isolates from *L. donovani*. This was done by combining downsampled read files from three cloned primary isolate/stock, ERR205809, ERR205816 and ERR205819, in various proportions (2.5, 5, 10, 15, 25 and 50% of the reads from the secondary clone), resulting in 33 MIX datasets, and comparing with 24 clones from Zackay 2018 [53]. We evaluated the two features that could impact the complexity estimations in multiclonal infections: the proportion of the secondary clone and the number of heterozygous SNP positions, using two parameters: the **CI**: which had to be higher than the mean + 3 standard deviations (SDEV) from the simulated clonal isolates in the population; and **CMH** test to evaluate if the real isolate AARD differs from the expected clonal isolate, with a p-value lower than 10^− 10^ (Fig. 2). Based on these cutoffs, zero clones (0%), and 26 (79%) of the MIX samples were classified as complex isolates. When evaluated separately, the CI parameter was the most specific, as only one clone was classified as complex (false positive), compared to 3 for CMH. However, CI was the least sensitive, as it only classified 26/33 (79%) MIX as complex, when compared to 31/33 (94%) for CMH (Fig. 2A and B, Supplementary Fig. 2, Additional File 9).

The CI accuracy was greatly influenced by the proportion of the secondary isolate, where lower proportions resulted in false negative results. None of the six MIX samples where the secondary clone read proportion was 2.5% was classified as complex. Increasing the proportion of the secondary clone resulted in higher accuracy, where five of the six samples where the secondary clone corresponded to 5% of the reads, and all samples where the secondary clone had 10–50% of the reads were classified as complex (Supplementary Fig. 2, Additional File 9). This was expected, as a low proportion of the secondary clone had a low impact in AARD distributions (Fig. 2B). Hence, our method can detect complex isolates when the secondary clone represents at least 5–10% of the reads.

To evaluate the impact of the number of heterozygous SNPs in the CI estimation, the heterozygous SNP counts for each MIX sample was downsampled to 10, 50, 100, 300 or full set, and the accuracy to classify each group as complex was assessed (Supplementary Fig. 2 I, Additional File 9). To remove potential SNP sampling bias, the analysis was repeated in 100 iterations, re-sampling random SNP positions each time, and the final results are a combination of all iterations. When compared with the full dataset, which had between 978 and 5910 SNPs, the use of 10 SNPs resulted in poor accuracy in all proportions of the secondary clone. By using 100 and 300 SNPs, the results were similar to those observed for the full set, with lower accuracy only for samples with ~ 5% of the reads originating from the secondary clone (Supplementary Fig. 2, Additional File 9; Supplementary Table 9, Additional File 10). Hence, the complexity index estimation requires 100 or more heterozygous SNPs to be accurate.

Besides multiclonality, another source of complexity is polyploidy, having extra full sets of chromosomal copies. To evaluate the impact of polyploidy in the complexity estimations, we used the *T. cruzi* dataset described in Matos 2022 [17], containing 8 diploid parental clones, and 11 triploid or tetraploid hybrids clones, where the somy of some were validated by flow cytometry Supplementary Table 3, Additional File 3. As performed for the multiclonal isolates, the SNPs counts for each sample were downsampled to 10, 50, 100, 300 or full set, in 100 iterations, and the accuracy to classify each group as complex was evaluated (Fig. 2C and D; and Supplementary Table 10, Additional File 11).

Using the combination of CI and CHM cutoffs, on average 4.4, 73.5, 82.5, 90.9 and 100% of the polyploid isolates, respectively for the 10, 50, 100, 300 or the full set of SNPs were correctly classified as complex. No parental diploid clones were classified as complex in any replicate. As expected, the triploid isolates had a distribution of AARD with peak distributions in 0.33 and 0.66, while the tetraploid had peaks in 0.25, 0.5 and 0.75. Both triploid and tetraploid isolates had an CI higher than the observed for the diploid isolates (Fig. 2C and D, Supplementary Fig. 3, Additional File 9). These results suggest that the complexity estimate can also be used to identify polyploid isolates with reasonable sensitivity (~ 80%) when 100 or more heterozygous SNPs are present.

Based on these results, we decided to use the combined results of CI and CHM tests to identify complex isolates, and to only evaluate samples with 100 or more SNPs. A conservative approach that minimises false positives, accepting some false negatives, especially in cases where the secondary clone proportion is low.

### Validating the CI accuracy to identify multiclonal infections with an independent dataset

To further validate the method accuracy in identifying multiclonal infections, we used 10 *T. cruzi* clones, including two pairs of clones that originated from the same primary isolate. We choose to further validate the method with *T. cruzi* as its genome is more repetitive than *Leishmania*, hence it is a challenging dataset for the method.

We generated tcMIX samples with all pairwise combinations of 5%, 10% and 20% of the secondary clones, and compared these with the results from “clones”, “same isolate clones” (two clones from the same primary isolate that were cultivated in different flasks) and “self samples” (combination of primary data and secondary data from the same clone). We identified 58%, 77% and 92% of the mixed isolates as complex or potential complex respectively for the 5%, 10% and 20% datasets. None of the clones, “same isolate clones” or “self samples” were classified as complex or potential complex (Supplementary Figs. 4 and 5, Additional File 9).

### Validating CI accuracy to identify polyploid hybrids isolates with independent datasets

To further validate the method accuracy in identifying polyploid isolates, we estimated the complexity of known *Leishmania* polyploid hybrids, described in Cata-Preta 2022 [55] and Louradour 2021 [54]. For the Cata-Preta 2023 dataset, we have correctly classified the 6 Triploid isolates as complex (LtHyb2, LtHyb3, LtHyb4, LtHyb5, LtHyb6, LtHyb7), and classified the Diploid hybrid LtHyb1 as well as the 6 diploid parental controls as non-complex (Supplementary Fig. 6, Additional File 9).

The Louradour dataset contains data from hybrids from three species: *L. braziliensis*; *L. tropica* and *L. donovani*. For the *L. braziliensis* dataset, we correctly classified the 9 tetraploid and one triploid isolate as complex, and the four diploid samples as non-complex (Supplementary Fig. 7, Additional File 9). For the *L. tropica* dataset, we correctly classified the five tetraploid and one triploid isolate as complex, and the seven diploid samples as non-complex (Supplementary Fig. 8, Additional File 9). Finally, for the *L. donovani* dataset, from the 17 tetraploid hybrids, we only classified one as complex (Supplementary Fig. 8, Additional File 9). After carefully evaluating this dataset, we believe that this was caused by the very low count of heterozygous SNPs and very high count of exclusive homozygous SNPs in each parental strain (Supplementary Fig. 9E, Additional File 9). This resulted in almost all of the heterozygous SNPs in the tetraploid hybrids to be generated by exclusive homozygous SNPs from each parent, resulting in a “balanced” AARD close to 0.5, as half of the chromosomes came from each parental strain. The unbalanced SNPs were also concentrated in a few chromosomes (ex: Chr1, 23 and part of Chr32), resulting in only 4–10% of the chromosomes having a CI higher than 0.1. Hence, the analysis of this dataset raised a limitation of the method: The identification of complexity in recent tetraploid hybrids, with no time to accumulate several individual mutations in each chromosome copy, from parentals that have significantly higher number of exclusive homozygous SNPs when compared to exclusive heterozygous SNPs. Similarly, a multiclonal infection with only two clones and 50% of each clone from clones that have significantly higher number of exclusive homozygous SNPs when compared to exclusive heterozygous SNPs may also be misclassified as non-complex. This limitation would not happen if the hybrids were triploid, or if the multiclonal isolate had an unbalanced proportion of each clone, or if the parental (or clones) had a significant number of exclusive heterozygous SNPs.

### Complexity evaluation among trypanosomatid species

**Fig. 3:**
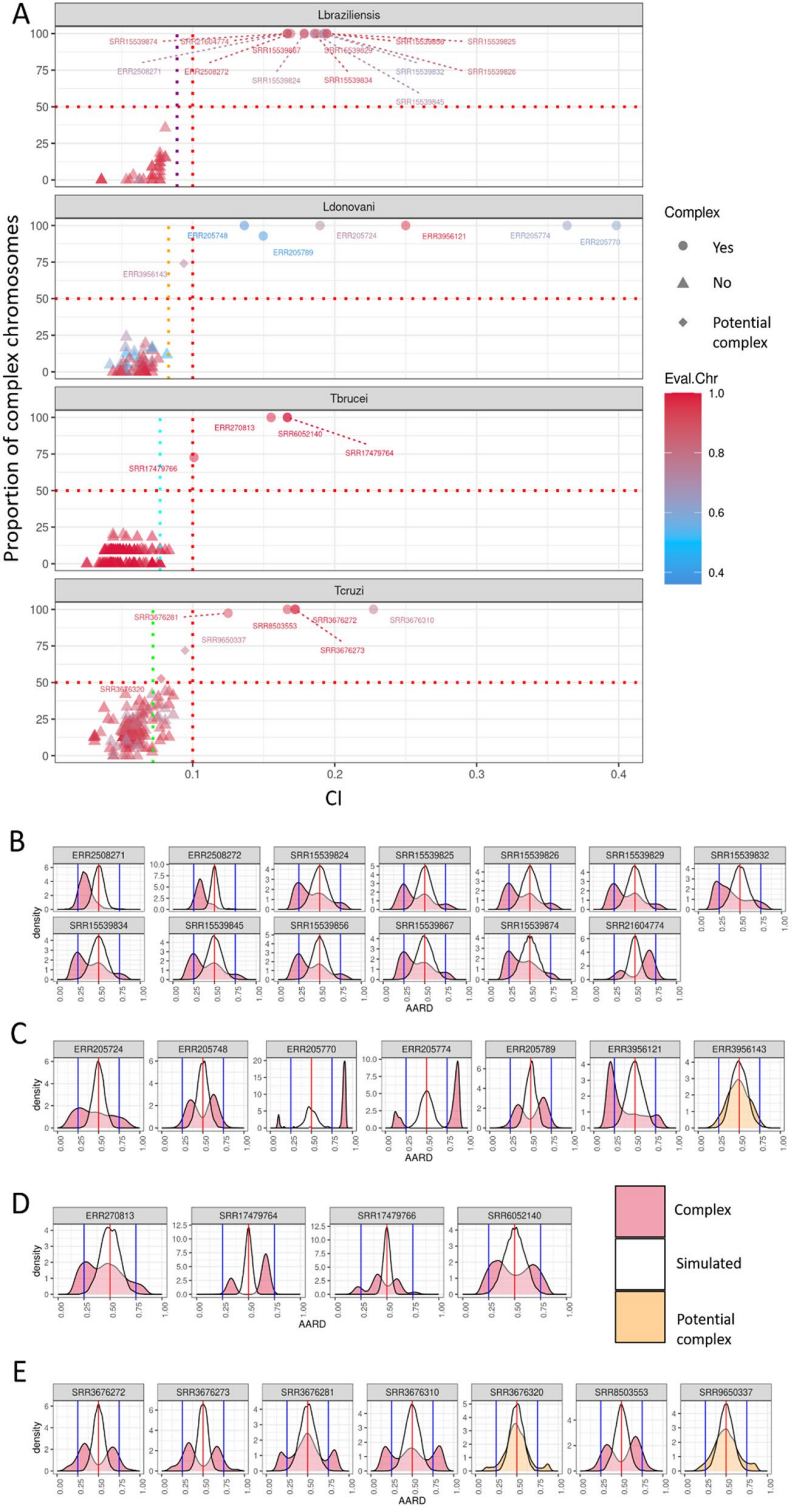
Overall complexity estimation in Trypanosomatids field isolates. (**A**) Complexity estimations in each sample from each species. Each box corresponds to a clade. From top to bottom: *L. braziliensis*, *L. donovani*, *T. brucei*, *T. cruzi*. Each dot corresponds to a complex (circles), potential complex (diamond) or non-complex (triangles) isolates. The X and Y axis represents, respectively, the CI and proportion of the evaluated chromosomes that had a CI ≥ 0.1. The colour corresponds to the proportion of chromosomes that were evaluated in the isolate. The vertical dotted lines represent complexity cutoffs, and are coloured in accordance to the species from whom they were estimated. The red vertical line is the global complexity cutoff of 0.1, which separates the potential complex from the complex isolates. (**B-E**) AARD distribution from the complex (red) and potential complex (orange) isolates. From top to bottom: *L. braziliensis*; *L. donovani*; *T. brucei*; *T. cruzi*. The distribution of the simulated clone is represented in white. The AARD density plot for all isolates from each population can be seen in the Supplementary Fig. 8 to 11, Additional File 9

After establishing the accuracy and limitations of the CI metric to identify multiclonal and polyploid samples with simulated and controlled data, we estimated the complexity in a total of 497 primary isolate/stock, clones and strains from *L. donovani*, *L. braziliensis*, *T. brucei* and *T. cruzi*, identifying a total of 28 complex isolates (Fig. 3; Table 1, Supplementary Figs. 10–13, Additional File 9). The CI cutoff was similar among the evaluated species, with the lowest value in *T. cruzi* (0.072) and the highest in *L. braziliensis* (0.089), which supports that the method should also work for other trypanosomatids and even potentially other diploid organisms. We propose a global cutoff of 0.1 (slightly higher than the highest cutoff, in *L. braziliensis*) as a value that may be used to classify any trypanosomatid isolate, or possibly other diploid eukaryotic samples, as complex, which will allow any researcher to classify single isolates without the need of population data to estimate a custom cutoff. Samples with CI values lower than the global cutoff but still higher than their species cutoff were classified as “potential complex” and evaluated separately. Only three potentially complex isolates were identified, two in *T. cruzi* and one in *L. donovani*.

**Table 1:** Complexity evaluation of each trypanosomatid group of samples

Species	Classification	Clone	Primary Isolate/Stock	Strain	Unknown	Assessment
*L. braziliensis* 42 samples	Complex (30%)	10	1	2	0	13 polyploid
	No	4	3	5	17	
*L. donovani* 85 samples	Complex (7%)	0	6	0	0	4 multiclonal 2 polyploid
	Pot.Complex	0	1	0	0	
	no	0	78	0	0	
*T. brucei* 159 samples	Complex (2.5%)	2	2	0	0	1 multiclonal 3 polyploid
	No	1	119	24	11	
*T. cruzi* 211 samples	Complex (2.3%)	0	5	0	0	2 multiclonal (chronic cases) 3 triploid (insect source)
	Pot. Complex	2	0	0	0	
	No	30	174	0	0	

The proportion of isolates that were classified as complex varied across clades, where *T. cruzi* and *T. brucei* had the lowest (~ 2.5%) and *L. braziliensis* had the highest (30%) proportion of complex samples in the evaluated dataset. Complex isolates have more heterozygous SNPs than non complex samples (Mann-Whitney p-value = 0.003), especially for *L. braziliensis* (Mann-Whitney p-value 2.87 × 10^− 6^) and *T. brucei* (Mann-Whitney, p-value 0.0074). This increase was not observed in the *L. donovani* evaluated samples (Supplementary Fig. 14, Additional File 9).

When each dataset was evaluated separately, from the 85 evaluated *L. donovani* samples, 6 primary isolate/stock were classified as complex (7%), and one as potentially complex. Among the 6 complex isolates, three ERR205724 (MHOM/SD/82/GILANI), ERR205770 (MHOM/IT/02/ISS2429) and ERR205774 (MHOM/BR/2003/MAM), were already classified as multiclonal by Fransen 2020 [59], and one, ERR3956121 (1052_ToD_1_primary_neg), was classified as complex by Frassen 2021 [31]. In fact, ERR205774 also presented a higher count of heterozygous SNPs in the maxicircle sequence, which further corroborates that it is a multiclonal infection (Supplementary Fig. 15, Additional File 9). Two isolates, ERR205748 (MHOM/CY/2006/CH32) and ERR205789 (MHOM/SD/62/LRC-L61), were classified by Frassen 2020 as hybrids, and had a ARRD distribution compatible with triploidy in our analysis. The sample that was classified as potential complex ERR3956143 (1073_ToD_1_primary_neg), corresponds to a primary isolate/Stock obtained from a patient from Ethiopia, which might be multiclonal.

For the *L. braziliensis* dataset, from the 42 evaluated samples, 13 (30%) were classified as complex and all had previous evidence of being polyploid. From these 13, 10 corresponded to experimental hybrids, described in [54] and previously used to assess the accuracy of somy variation estimates; while SRR21604774 corresponded to a triploid *L. braziliensis* and *Leishmania guyanensis* hybrid. Finally the last two samples, ERR2508271 and ERR2508272, correspond to read libraries used in the assembly of the triploid *L. braziliensis* M2904 genome [16, 56]. We found no strong evidence of multiclonal infections in any of the evaluated *L. braziliensis* samples.

From the 211 *T. cruzi* evaluated samples, five primary isolate/stock were classified as complex, and two clones were classified as potential complex. From the complex samples, three were isolated from the insect vector: SRR8503553 (*Panstrongylus lignarius* in Peru); SRR3676272 and SRR3676273 (*Triatoma dimidiata* in Texas) [70, 77, 78]. The AARD density peaks in these three samples are similar to those expected for triploid isolates (0.33 and 0.66), suggesting that they are polyploid. The other two complex samples were isolated from chronic chagasic human patients in Panama (SRR3676281, SRR3676310) [70], and had AARD peaks that are not similar to what is expected for tri or tetraploid isolates. This suggests that they might be multiclonal infections. In fact, SRR3676310 had also a higher count of heterozygous SNPs in the maxicircle sequence when compared to other *T. cruzi* isolates (Supplementary Fig. 15, Additional File 9), which further support that it is potentially a multiclonal infection.

For *T. brucei* we identified 4 complex isolates in a dataset of 159 samples. From those two were clones: SRR17479764 corresponds to a triploid hybrid from the J10 and KETRI 1738 strains [60], while SRR17479766 (F1R1) was previously suggested to be a multiclonal infection, even being cloned [60]. The final two complex *T. brucei* strains, ERR270813 and SRR6052140 are primary isolate/stocks that have AARD profiles that are similar to what is respectively expected for tetraploid and triploid isolates.

Finally, to assess the complexity of intra-species hybrids, we have evaluated the *Leishmania* Sri Lanka primary isolate/stocks described in Lypaczewski 2021 [34]. From the seven samples that had coverage above 25 and more than 100 SNPs, 5 were classified as complex and two as non-complex (Supplementary Fig. 16, Additional File 9). For the samples classified as complex, the AARD was skewed to 0.25 (SRR6257364, SRR6257365, SRR6257369), 0.25 and 0.5 (SRR6257371) or 0.66 (SRR6257366), which suggests respectively tetraploid and triploid hybrids. Four of the five complex isolates (SRR6257364, SRR6257365, SRR6257366 and SRR6257369) were classified on the SL2 sub-group by Lypaczewski 2021, which the authors have also observed deviations from the 0.5 AARD and suggested that are characterised by recent hybrids. The two strains that were classified as non-complex in our analysis, SRR6257368 and SRR6257370, and one that were classified as complex, SRR6257371, were classified by Lypaczewski [34] in SL3, which they suggest is an ancient hybridization event. Hence, samples from this group might have had more time to revert to disomy after hybridization.

Taken together these results suggest that complex isolates represent a small percentage of the primary isolate/stock and strains for all the TriTryp species evaluated. This corresponds to the lower bound of potential complex infections compared to what is observed in natural conditions due to limitations in parasite isolation, culture and a low proportion of the secondary clone, which hampers complexity detection by our method. We provide R code for CI estimation on github (https://github.com/jaumlrc/Complex-Infections.git).

### Discussion

In the present study, we identified and characterised two features that result in more than two haplotypes being present in a single parasite isolate in trypanosomatids: multiclonal infections and polyploidy. We developed and validated a method to assess the complexity of trypanosomatid samples based on WGS reads, and implemented the method to evaluate complexity in a representative collection of *Leishmania*, *T. cruzi* and *T. brucei* primary isolate/stock, clones and strains. Our method only uses chromosomes with similar somy as the genome ploidy, and removes genes with evidence of duplication/loss, as these could be confounding factors in the estimations. We have identified complex (polyploidy or multiclonal) infections in all evaluated species, and proposed a global complexity index cutoff that can be used in any trypanosomatid single sample, and likely other diploid eukaryote samples.

In the last decade, the reduction in sequencing costs and the relevance of questions that may be answered with genomic data have resulted in a large increase in the number of studies that generate population WGS data for trypanosomatid parasites [31, 53, 59, 61, 70, 72, 76, 79–81]. However, the occurrence of multiclonal infection and polyploidy is not always assessed in these studies. Complex infections also occur in bacterial infections, viruses and some protozoan parasites as *Plasmodium*, where the main stage that infects humans and other mammalian hosts is haploid [82, 83]. Trypanosomatid parasites are usually diploid, and often aneuploid and/or polyploid [16, 20, 76, 84], where the somy of different chromosomes can vary even within clones [73]. This increases the challenge of estimating complex infections in these parasites. Hence, a method is needed to identify complex infections using WGS in these species at scale, that considers gene copy number variants and aneuploidy.

By using WGS reads, the method that we propose has the advantage of assessing genome-wide SNP variation as evidence for complexity [22, 59]. When compared to methods based on microsatellite loci and marker genes [23–25, 27], the use of WGS data allows the removal of aneuploid chromosomes and duplicated genes based on read depth values. By evaluating the complexity in each euploid chromosome from an isolate and only classifying as complex samples that have complexity evidence supported by at least half of the evaluated chromosomes, we could also mitigate the impact of “chromosome instability” (CIN) and “mosaic aneuploidy” events [16, 73, 75, 76].

In the current study, we identified a low proportion of complex infections in all trypanosomatid primary isolate/stock and strains. We identified around 7% of complex infections for the *L. donovani* group, in accordance with what was identified in [23], where even though different *Leishmania* genotypes were identified in different tissues, the number of isolates with MOI in the same tissue was low. For *T. cruzi*, we identified a very low proportion of complex infections (~ 2%), which is lower than the ~ 15–17% that was reported in the literature for inter Discrete Typing Units (DTU) [85] mixed infections in human patients from Latin America [24, 86, 87]; and to the ~ 13% of MOI in the vector *Triatoma infestans* [88]. This might be caused in part as ~ 15% of the evaluated *T. cruzi* samples were cloned prior to sequencing [72]. As cloned samples could be polyploid, they were still evaluated in this work. Although most of the *T. cruzi* isolates had an AARD distribution that matched the expectation from a “non-complex clonal, euploid, diploid isolate”, there were some non-complex isolates with perturbations in the AARD distribution and high CI; such as SRR3676315, SRR3676316, SRR3676317, SRR3676318, SRR3676319; that had a distribution pattern similar to the potential complex isolate SRR3676320. These isolates had a high CI in less than half of the evaluated chromosomes, which suggests that they have a high level of mosaic aneuploidy and CIN [20, 71, 75, 76]. *T. brucei* isolates also had a low proportion of complex infections identified in the WGS data (~ 2%) with only one isolate with strong evidence of multiclonal infection, which is lower than the 8–20% of multiclonal infections reported in humans and vector infections in East Africa [27].

The proposed method was able to identify complex infections when the secondary clone corresponded to at least 5–10% of the sequencing reads. This is in the range of what was observed in WGS from clinical samples with Sure-select sequencing, where in the three identified complex infections, the proportion of the secondary clone was ~ 6–10% [22]. However, the mean genome coverage of the primary isolate/stock and strains evaluated here varied from 29 to 56 among the datasets, limiting our potential to identify multiclonal infections to cases where the secondary clone corresponded to at least ~ 8–17% of the total reads in the sample (Supplementary Fig. 14G, Additional File 9).

Potential limitations of complexity estimation based on WGS are data collection, processing and genome coverage. Most trypanosomatid WGS data is obtained from parasites that are isolated from the host and cultured in axenic media or used to infect mice, which might reduce complexity when compared to the variation present in the patient [22, 23].

There is evidence of some degree of sexual recombination and outcrossing in the three evaluated trypanosomatid groups [29], indicating that different genotypes must be present in the same insect at some point to undergo meiosis and outcrossing. Bottlenecks in the number of parasites transferred to or from vectors may reduce genetic diversity of infections at the outset, and long incubation of parasites within mammalian hosts with selection for the fittest parasite genotype may reduce genetically complex populations to a single clone. A reduction in within-host diversity would be expected to reduce outcrossing, unless vectors frequently feed on more than one host. We can expect that there will be alternative explanations for different species and populations, depending on the frequencies of transmission, endemicity and within host/within vector population dynamics. It is our perspective that more study of these factors will enhance our understanding of transmission dynamics in trypanosomatids.

## Conclusions

The method we describe can accurately identify polyploid and multiclonal infections in samples sequenced with modest read depth (> 25x), as little as 100 heterozygous SNPs, and as little as 5–10% of the secondary genotype. We find that multiclonality and polyploidy are not frequent in cultured trypanosomatid primary isolate/stock, although there are good reasons to expect that our estimates are lower bounds. Future projects could explore new sequencing methods to identify multiclonal infections, such as single-cell sequencing [89] that could directly identify different clones; and long-read sequencing followed by haplotype phasing, to identify different haplotypes in a sample [90–92]. These methods could quantify the proportion and number of the different clones in a mixed infection.

## Electronic supplementary material

Below is the link to the electronic supplementary material.

Supplementary Material 1: *L. donovani* clones and MIX samples used in the initial multiclonal complexity assessment.

Supplementary Material 2: *T. cruzi* clones and MIX samples used in the multiclonal complexity validation.

Supplementary Material 3: *T. cruzi* clones and hybrid samples used in the initial polyploidy complexity assessment.

Supplementary Material 4: *Leishmania* clones and hybrid samples used in the initial polyploidy complexity assessment from Louradour 2021.

Supplementary Material 5: *Leishmania* clones and hybrid samples used in the initial polyploidy complexity assessment from Cata-Preta 2022.

Supplementary Material 6: Trypanosomatid primary isolates, clones and stocks used in complexity assessment.

Supplementary Material 7: *Leishmania* hybrids from Sri Lanka used in complexity assessment, described in Lypaczewski 2021.

Supplementary Material 8: Reference genomes used in read mapping.

Supplementary Material 9: Contains all the 16 supplementary figures.

Supplementary Material 10: Evaluation of the impact of SNP counts on complexity estimations for the MIX samples.

Supplementary Material 11: Evaluation of the impact of SNP counts on complexity estimations for the polyploid samples.

## Data Availability

All the read libraries used in this are available in NCBI (see Supplementary Tables 1 to 7). The script and test set can be obtained from GitHub: https://github.com/jaumlrc/Complex-Infections.git.

## References

[1] Burza S, Croft SL, Boelaert M, Leishmaniasis. Lancet. 2018;392:951–70. 10.1016/S0140-6736(18)31204-2 30126638

[2] Kennedy PGE. Update on human African trypanosomiasis (sleeping sickness). J Neurol. 2019;266:2334–7. 31209574 10.1007/s00415-019-09425-7

[3] Horn D. A profile of research on the parasitic trypanosomatids and the diseases they cause. PLoS Negl Trop Dis. 2022;16:e0010040. 35025891 10.1371/journal.pntd.0010040 PMC8758061

[4] Vickerman K. Antigenic variation in trypanosomes. Nature. 1978;273:613–7. 661969 10.1038/273613a0

[5] Horn D. Antigenic variation in African trypanosomes. Mol Biochem Parasitol. 2014;195:123–9. 24859277 10.1016/j.molbiopara.2014.05.001 PMC4155160

[6] Stockdale C, Swiderski MR, Barry JD, McCulloch R. Antigenic variation in *Trypanosoma Brucei*: joining the DOTs. PLoS Biol. 2008;6:e185. 18666832 10.1371/journal.pbio.0060185 PMC2486309

[7] Faria J, Briggs EM, Black JA, McCulloch R. Emergence and adaptation of the cellular machinery directing antigenic variation in the African trypanosome. Curr Opin Microbiol. 2022;70:102209. 36215868 10.1016/j.mib.2022.102209

[8] De Pablos LM, Osuna A. Multigene families in *Trypanosoma Cruzi* and their role in infectivity. Infect Immun. 2012;80:2258–64. 22431647 10.1128/IAI.06225-11 PMC3416482

[9] El-Sayed NM, Myler PJ, Bartholomeu DC, Nilsson D, Aggarwal G, Tran A-N, et al. The genome sequence of *Trypanosoma Cruzi*, etiologic agent of Chagas disease. Science. 2005;309:409–15. 16020725 10.1126/science.1112631

[10] Herreros-Cabello A, Callejas-Hernández F, Gironès N, Fresno M. *Trypanosoma cruzi* Genome: Organization, Multi-Gene Families, Transcription, and Biological Implications. Genes. 2020;11. 10.3390/genes11101196 PMC7602482 33066599

[11] Gupta G, Oghumu S, Satoskar AR. Mechanisms of immune evasion in leishmaniasis. Adv Appl Microbiol. 2013;82:155–84. 23415155 10.1016/B978-0-12-407679-2.00005-3 PMC3697132

[12] Cardoso MS, Reis-Cunha JL, Bartholomeu DC. Evasion of the Immune response by *Trypanosoma Cruzi* during Acute infection. Front Immunol. 2015;6:659. 26834737 10.3389/fimmu.2015.00659 PMC4716143

[13] Fernandes MC, Andrews NW. Host cell invasion by *Trypanosoma Cruzi*: a unique strategy that promotes persistence. FEMS Microbiol Rev. 2012;36:734–47. 22339763 10.1111/j.1574-6976.2012.00333.x PMC3319478

[14] Reis-Cunha JL, Pimenta-Carvalho SA, Almeida LV, Coqueiro-Dos-Santos A, Marques CA, Black JA, et al. Ancestral aneuploidy and stable chromosomal duplication resulting in differential genome structure and gene expression control in trypanosomatid parasites. Genome Res. 2024;34:441–53. 38604731 10.1101/gr.278550.123 PMC11067883

[15] Dumetz F, Imamura H, Sanders M, Seblova V, Myskova J, Pescher P et al. Modulation of Aneuploidy in *Leishmania donovani* during adaptation to different in Vitro and in vivo environments and its impact on Gene expression. MBio. 2017;8. 10.1128/mBio.00599-17 PMC5442457 28536289

[16] Rogers MB, Hilley JD, Dickens NJ, Wilkes J, Bates PA, Depledge DP, et al. Chromosome and gene copy number variation allow major structural change between species and strains of *Leishmania*. Genome Res. 2011;21:2129–42. 22038252 10.1101/gr.122945.111 PMC3227102

[17] Matos GM, Lewis MD, Talavera-López C, Yeo M, Grisard EC, Messenger LA et al. Microevolution of *Trypanosoma Cruzi* reveals hybridization and clonal mechanisms driving rapid genome diversification. Elife. 2022;11. 10.7554/eLife.75237 PMC9098224 35535495

[18] Louradour I, Ferreira TR, Ghosh K, Shaik J, Sacks D. In Vitro Generation of *Leishmania* hybrids. Cell Rep. 2020;31:107507. 32294444 10.1016/j.celrep.2020.03.071

[19] Tihon E, Imamura H, Dujardin J-C, Van Den Abbeele J. Evidence for viable and stable triploid *Trypanosoma congolense* parasites. Parasit Vectors. 2017;10:468. 29017575 10.1186/s13071-017-2406-z PMC5635536

[20] Black JA, Reis-Cunha JL, Cruz AK, Tosi LRO. Life in plastic, it’s fantastic! How *Leishmania* exploit genome instability to shape gene expression. Front Cell Infect Microbiol. 2023;13:1102462. 36779182 10.3389/fcimb.2023.1102462 PMC9910336

[21] Balmer O, Tanner M. Prevalence and implications of multiple-strain infections. Lancet Infect Dis. 2011;11:868–78. 22035615 10.1016/S1473-3099(11)70241-9

[22] Domagalska MA, Imamura H, Sanders M, Van den Broeck F, Bhattarai NR, Vanaerschot M, et al. Genomes of *Leishmania* parasites directly sequenced from patients with visceral leishmaniasis in the Indian subcontinent. PLoS Negl Trop Dis. 2019;13:e0007900. 31830038 10.1371/journal.pntd.0007900 PMC6932831

[23] Cupolillo E, Cavalcanti AS, Ferreira GEM, Boité MC, Morgado FN, Porrozzi R. Occurrence of multiple genotype infection caused by *Leishmania infantum* in naturally infected dogs. PLoS Negl Trop Dis. 2020;14:e0007986. 32716941 10.1371/journal.pntd.0007986 PMC7410330

[24] Martinez-Perez A, Poveda C, Ramírez JD, Norman F, Gironés N, Guhl F, et al. Prevalence of *Trypanosoma Cruzi*’s discrete typing units in a cohort of latin American migrants in Spain. Acta Trop. 2016;157:145–50. 26851167 10.1016/j.actatropica.2016.01.032

[25] Llewellyn MS, Rivett-Carnac JB, Fitzpatrick S, Lewis MD, Yeo M, Gaunt MW, et al. Extraordinary *Trypanosoma Cruzi* diversity within single mammalian reservoir hosts implies a mechanism of diversifying selection. Int J Parasitol. 2011;41:609–14. 21232539 10.1016/j.ijpara.2010.12.004 PMC3084450

[26] Pronovost H, Peterson AC, Chavez BG, Blum MJ, Dumonteil E, Herrera CP. Deep sequencing reveals multiclonality and new discrete typing units of *Trypanosoma Cruzi* in rodents from the southern United States. J Microbiol Immunol Infect. 2020;53:622–33. 30709717 10.1016/j.jmii.2018.12.004

[27] Balmer O, Caccone A. Multiple-strain infections of *Trypanosoma Brucei* across Africa. Acta Trop. 2008;107:275–9. 18671933 10.1016/j.actatropica.2008.06.006 PMC2582348

[28] Bose J, Kloesener MH, Schulte RD. Multiple-genotype infections and their complex effect on virulence. Zoology. 2016;119:339–49. 27389395 10.1016/j.zool.2016.06.003

[29] Gutiérrez-Corbo C, Domínguez-Asenjo B, Martínez-Valladares M, Pérez-Pertejo Y, García-Estrada C, Balaña-Fouce R et al. Reprod Trypanosomatids: Past Present Biology. 2021;10. 10.3390/biology10060471 PMC8230138 34071741

[30] Read AF, Taylor LH. The ecology of genetically diverse infections. Science. 2001;292:1099–102. 11352063 10.1126/science.1059410

[31] Franssen SU, Takele Y, Adem E, Sanders MJ, Müller I, Kropf P, et al. Diversity and within-host evolution of *Leishmania donovani* from visceral leishmaniasis patients with and without HIV Coinfection in Northern Ethiopia. MBio. 2021;12:e0097121. 34182785 10.1128/mBio.00971-21 PMC8262925

[32] Darvishi M, Yaghoobi-Ershadi MR, Shahbazi F, Akhavan AA, Jafari R, Soleimani H, et al. Epidemiological study on sand flies in an endemic focus of cutaneous leishmaniasis, Bushehr city, southwestern Iran. Front Public Health. 2015;3:14. 25699245 10.3389/fpubh.2015.00014 PMC4313593

[33] Chajbullinova A, Votypka J, Sadlova J, Kvapilova K, Seblova V, Kreisinger J, et al. The development of *Leishmania Turanica* in sand flies and competition with *L. Major*. Parasit Vectors. 2012;5:219. 23031344 10.1186/1756-3305-5-219 PMC3484061

[34] Lypaczewski P, Matlashewski G. *LeishDonovaninovani* hybridisation and introgression in nature: a comparative genomic investigation. Lancet Microbe. 2021;2:e250–8. 35544170 10.1016/S2666-5247(21)00028-8

[35] MacLeod A, Turner CM, Tait A. A high level of mixed *Trypanosoma brucei* infections in tsetse flies detected by three hypervariable minisatellites. Mol Biochem Parasitol. 1999;102:237–48. 10498180 10.1016/s0166-6851(99)00101-2

[36] Balmer O, Stearns SC, Schötzau A, Brun R. Intraspecific competition between co-infecting parasite strains enhances host survival in African trypanosomes. Ecology. 2009;90:3367–78. 20120806 10.1890/08-2291.1

[37] Dumonteil E, Desale H, Tu W, Hernandez-Cuevas N, Shroyer M, Goff K, et al. Intra-host *Trypanosoma Cruzi* strain dynamics shape disease progression: the missing link in Chagas disease pathogenesis. Microbiol Spectr. 2023;11:e0423622. 37668388 10.1128/spectrum.04236-22 PMC10581044

[38] Shirian S, Oryan A, Hatam GR, Daneshbod Y. Mixed mucosal leishmaniasis infection caused by *Leishmania Tropica* and *Leishmania major*. J Clin Microbiol. 2012;50:3805–8. 22972819 10.1128/JCM.01469-12 PMC3486260

[39] Ferreira TR, Sacks DL. Experimental hybridization in *Leishmania*: tools for the study of Genetic Exchange. Pathogens. 2022;11. 10.3390/pathogens11050580 PMC9144296 35631101

[40] Akopyants NS, Kimblin N, Secundino N, Patrick R, Peters N, Lawyer P, et al. Demonstration of genetic exchange during cyclical development of *Leishmania* in the sand fly vector. Science. 2009;324:265–8. 19359589 10.1126/science.1169464 PMC2729066

[41] Inbar E, Akopyants NS, Charmoy M, Romano A, Lawyer P, Elnaiem D-EA, et al. The mating competence of geographically diverse *Leishmania major* strains in their natural and unnatural sand fly vectors. PLoS Genet. 2013;9:e1003672. 23935521 10.1371/journal.pgen.1003672 PMC3723561

[42] Romano A, Inbar E, Debrabant A, Charmoy M, Lawyer P, Ribeiro-Gomes F, et al. Cross-species genetic exchange between visceral and cutaneous strains of *Leishmania* in the sand fly vector. Proc Natl Acad Sci U S A. 2014;111:16808–13. 25385616 10.1073/pnas.1415109111 PMC4250153

[43] Pritchard JK, Stephens M, Donnelly P. Inference of population structure using multilocus genotype data. Genetics. 2000;155:945–59. 10835412 10.1093/genetics/155.2.945 PMC1461096

[44] Browning BL, Tian X, Zhou Y, Browning SR. Fast two-stage phasing of large-scale sequence data. Am J Hum Genet. 2021;108:1880–90. 34478634 10.1016/j.ajhg.2021.08.005 PMC8551421

[45] Alexander DH, Novembre J, Lange K. Fast model-based estimation of ancestry in unrelated individuals. Genome Res. 2009;19:1655–64. 19648217 10.1101/gr.094052.109 PMC2752134

[46] Long Q, Jeffares DC, Zhang Q, Ye K, Nizhynska V, Ning Z, et al. PoolHap: inferring haplotype frequencies from pooled samples by next generation sequencing. PLoS ONE. 2011;6:e15292. 21264334 10.1371/journal.pone.0015292 PMC3016441

[47] Nucleotide Sequence I. The sequence read archive. Nucleic acids. 2010.

[48] Chen S, Zhou Y, Chen Y, Gu J. Fastp: an ultra-fast all-in-one FASTQ preprocessor. Bioinformatics. 2018;34:i884–90. 30423086 10.1093/bioinformatics/bty560 PMC6129281

[49] Li H. Aligning sequence reads, clone sequences and assembly contigs with BWA-MEM. arXiv [q-bio.GN]. 2013.

[50] Li H, Handsaker B, Wysoker A, Fennell T, Ruan J, Homer N, et al. The sequence Alignment/Map format and SAMtools. Bioinformatics. 2009;25:2078–9. 19505943 10.1093/bioinformatics/btp352 PMC2723002

[51] Emms DM, Kelly S. OrthoFinder: phylogenetic orthology inference for comparative genomics. Genome Biol. 2019;20:238. 31727128 10.1186/s13059-019-1832-y PMC6857279

[52] Danecek P, Bonfield JK, Liddle J, Marshall J, Ohan V, Pollard MO et al. Twelve years of SAMtools and BCFtools. Gigascience. 2021;10. 10.1093/gigascience/giab008 PMC7931819 33590861

[53] Zackay A, Cotton JA, Sanders M, Hailu A, Nasereddin A, Warburg A, et al. Genome wide comparison of Ethiopian *Leishmania donovani* strains reveals differences potentially related to parasite survival. PLoS Genet. 2018;14:e1007133. 29315303 10.1371/journal.pgen.1007133 PMC5777657

[54] Louradour I, Ferreira TR, Duge E, Karunaweera N, Paun A, Sacks D. Stress conditions promote *Leishmania* hybridization in vitro marked by expression of the ancestral gamete fusogen HAP2 as revealed by single-cell RNA-seq. Elife. 2022;11. 10.7554/eLife.73488 PMC8794473 34994687

[55] Catta-Preta CMC, Ferreira TR, Ghosh K, Paun A, Sacks D. HOP1 and HAP2 are conserved components of the meiosis-related machinery required for successful mating in *Leishmania*. Nat Commun. 2023;14:7159. 37935664 10.1038/s41467-023-42789-z PMC10630298

[56] González-de la Fuente S, Camacho E, Peiró-Pastor R, Rastrojo A, Carrasco-Ramiro F, Aguado B, et al. Complete and de novo assembly of the *Leishmania braziliensis* (M2904) genome. Mem Inst Oswaldo Cruz. 2018;114:e180438. 30540030 10.1590/0074-02760180438 PMC6319030

[57] Van den Broeck F, Heeren S, Maes I, Sanders M, Cotton JA, Cupolillo E, et al. Genome Analysis of Triploid Hybrid *Leishmania* Parasite from the Neotropics. Emerg Infect Dis. 2023;29:1076–8. 37081624 10.3201/eid2905.221456 PMC10124652

[58] Imamura H, Downing T, Van den Broeck F, Sanders MJ, Rijal S, Sundar S et al. Evolutionary genomics of epidemic visceral leishmaniasis in the Indian subcontinent. Elife. 2016;5. 10.7554/eLife.12613 PMC4811772 27003289

[59] Franssen SU, Durrant C, Stark O, Moser B, Downing T, Imamura H et al. Global genome diversity of the *Leishmania Donovani* complex. Elife. 2020;9. 10.7554/eLife.51243 PMC7105377 32209228

[60] Kay C, Peacock L, Williams TA, Gibson W. Signatures of hybridization in *Trypanosoma Brucei*. PLoS Pathog. 2022;18:e1010300. 35139131 10.1371/journal.ppat.1010300 PMC8863249

[61] Weir W, Capewell P, Foth B, Clucas C, Pountain A, Steketee P, et al. Population genomics reveals the origin and asexual evolution of human infective trypanosomes. Elife. 2016;5:e11473. 26809473 10.7554/eLife.11473 PMC4739771

[62] Cosentino RO, Brink BG, Siegel TN. Allele-specific assembly of a eukaryotic genome corrects apparent frameshifts and reveals a lack of nonsense-mediated mRNA decay. NAR Genom Bioinform. 2021;3:lqab082. 34541528 10.1093/nargab/lqab082 PMC8445201

[63] Cooper S, Wadsworth ES, Ochsenreiter T, Ivens A, Savill NJ, Schnaufer A. Assembly and annotation of the mitochondrial minicircle genome of a differentiation-competent strain of *Trypanosoma Brucei*. Nucleic Acids Res. 2019;47:11304–25. 31665448 10.1093/nar/gkz928 PMC6868439

[64] Cuypers B, Van den Broeck F, Van Reet N, Meehan CJ, Cauchard J, Wilkes JM, et al. Genome-wide SNP analysis reveals distinct origins of *Trypanosoma Evansi* and *Trypanosoma Equiperdum*. Genome Biol Evol. 2017;9:1990–7. 28541535 10.1093/gbe/evx102 PMC5566637

[65] Davaasuren B, Yamagishi J, Mizushima D, Narantsatsral S, Otgonsuren D, Myagmarsuren P et al. Draft genome sequence of *Trypanosoma Equiperdum* strain IVM-t1. Microbiol Resour Announc. 2019;8. 10.1128/MRA.01119-18 PMC6395869 30834384

[66] Devlin R, Marques CA, Paape D, Prorocic M, Zurita-Leal AC, Campbell SJ et al. Mapping replication dynamics in *Trypanosoma Brucei* reveals a link with telomere transcription and antigenic variation. Elife. 2016;5. 10.7554/eLife.12765 PMC4946898 27228154

[67] Giordani F, Paape D, Vincent IM, Pountain AW, Fernández-Cortés F, Rico E, et al. Veterinary trypanocidal benzoxaboroles are peptidase-activated prodrugs. PLoS Pathog. 2020;16:e1008932. 33141865 10.1371/journal.ppat.1008932 PMC7710103

[68] Mulindwa J, Ssentamu G, Matovu E, Kamanyi Marucha K, Aresta-Branco F, Helbig C, et al. In vitro culture of freshly isolated *Trypanosoma Brucei Brucei* bloodstream forms results in gene copy-number changes. PLoS Negl Trop Dis. 2021;15:e0009738. 34516555 10.1371/journal.pntd.0009738 PMC8459984

[69] Berry ASF, Salazar-Sánchez R, Castillo-Neyra R, Borrini-Mayorí K, Arevalo-Nieto C, Chipana-Ramos C, et al. Dispersal patterns of *Trypanosoma Cruzi* in Arequipa, Peru. PLoS Negl Trop Dis. 2020;14:e0007910. 32150562 10.1371/journal.pntd.0007910 PMC7082062

[70] Talavera-López C, Messenger LA, Lewis MD, Yeo M, Reis-Cunha JL, Matos GM, et al. Repeat-driven generation of antigenic diversity in a Major Human Pathogen, *Trypanosoma Cruzi*. Front Cell Infect Microbiol. 2021;11:614665. 33747978 10.3389/fcimb.2021.614665 PMC7966520

[71] Reis-Cunha JL, Valdivia HO, Bartholomeu DC. Gene and Chromosomal Copy Number variations as an adaptive mechanism towards a parasitic lifestyle in Trypanosomatids. Curr Genomics. 2018;19:87–97. 29491737 10.2174/1389202918666170911161311 PMC5814966

[72] Schwabl P, Imamura H, Van den Broeck F, Costales JA, Maiguashca-Sánchez J, Miles MA, et al. Meiotic sex in Chagas disease parasite *Trypanosoma Cruzi*. Nat Commun. 2019;10:3972. 31481692 10.1038/s41467-019-11771-z PMC6722143

[73] Negreira GH, Monsieurs P, Imamura H, Maes I, Kuk N, Yagoubat A, et al. High throughput single-cell genome sequencing gives insights into the generation and evolution of mosaic aneuploidy in *Leishmania donovani*. Nucleic Acids Res. 2022;50:293–305. 34893872 10.1093/nar/gkab1203 PMC8886908

[74] Negreira GH, de Groote R, Van Giel D, Monsieurs P, Maes I, de Muylder G, et al. The adaptive roles of aneuploidy and polyclonality in *Leishmania* in response to environmental stress. EMBO Rep. 2023;24:e57413. 37470283 10.15252/embr.202357413 PMC10481652

[75] Lachaud L, Bourgeois N, Kuk N, Morelle C, Crobu L, Merlin G, et al. Constitutive mosaic aneuploidy is a unique genetic feature widespread in the *Leishmania* Genus. Microbes Infect. 2014;16:61–6. 24120456 10.1016/j.micinf.2013.09.005

[76] Reis-Cunha JL, Rodrigues-Luiz GF, Valdivia HO, Baptista RP, Mendes TAO, de Morais GL, et al. Chromosomal copy number variation reveals differential levels of genomic plasticity in distinct *Trypanosoma Cruzi* strains. BMC Genomics. 2015;16:499. 26141959 10.1186/s12864-015-1680-4 PMC4491234

[77] Berry ASF, Salazar-Sánchez R, Castillo-Neyra R, Borrini-Mayorí K, Chipana-Ramos C, Vargas-Maquera M, et al. Immigration and establishment of *Trypanosoma Cruzi* in Arequipa, Peru. PLoS ONE. 2019;14:e0221678. 31454370 10.1371/journal.pone.0221678 PMC6711515

[78] Berry ASF, Salazar-Sánchez R, Castillo-Neyra R, Borrini-Mayorí K, Chipana-Ramos C, Vargas-Maquera M, et al. Sexual reproduction in a natural *Trypanosoma Cruzi* population. PLoS Negl Trop Dis. 2019;13:e0007392. 31107905 10.1371/journal.pntd.0007392 PMC6544315

[79] Reis-Cunha JL, Baptista RP, Rodrigues-Luiz GF, Coqueiro-Dos-Santos A, Valdivia HO, de Almeida LV, et al. Whole genome sequencing of *Trypanosoma Cruzi* field isolates reveals extensive genomic variability and complex aneuploidy patterns within TcII DTU. BMC Genomics. 2018;19:816. 30424726 10.1186/s12864-018-5198-4 PMC6234542

[80] Almeida LV, Coqueiro-Dos-Santos A, Rodriguez-Luiz GF, McCulloch R, Bartholomeu DC, Reis-Cunha JL. Chromosomal copy number variation analysis by next generation sequencing confirms ploidy stability in *Trypanosoma Brucei* subspecies. Microb Genom. 2018;4. 10.1099/mgen.0.000223 PMC6249438 30256189

[81] Grace CA, Sousa Carvalho KS, Sousa Lima MI, Costa Silva V, Reis-Cunha JL, Brune MJ, et al. Parasite genotype is a major predictor of mortality from visceral leishmaniasis. MBio. 2022;13:e0206822. 36222512 10.1128/mbio.02068-22 PMC9765272

[82] Assefa SA, Preston MD, Campino S, Ocholla H, Sutherland CJ, Clark TG. estMOI: estimating multiplicity of infection using parasite deep sequencing data. Bioinformatics. 2014;30:1292–4. 24443379 10.1093/bioinformatics/btu005 PMC3998131

[83] Zhong D, Koepfli C, Cui L, Yan G. Molecular approaches to determine the multiplicity of Plasmodium infections. Malar J. 2018;17:172. 29685152 10.1186/s12936-018-2322-5 PMC5914063

[84] Reis-Cunha JL, Valdivia HO, Bartholomeu DC. Trypanosomatid Genome Organization and Ploidy. Front Parasitol. 2017;:61–103.

[85] Velásquez-Ortiz N, Herrera G, Hernández C, Muñoz M, Ramírez JD. Discrete typing units of *Trypanosoma Cruzi*: geographical and biological distribution in the Americas. Sci Data. 2022;9:360. 35750679 10.1038/s41597-022-01452-w PMC9232490

[86] Perez-Molina JA, Poveda C, Martinez-Perez A, Guhl F, Monge-Maillo B, Fresno M, et al. Distribution of *Trypanosoma Cruzi* discrete typing units in Bolivian migrants in Spain. Infect Genet Evol. 2014;21:440–2. 24389118 10.1016/j.meegid.2013.12.018

[87] Cura CI, Lucero RH, Bisio M, Oshiro E, Formichelli LB, Burgos JM, et al. *TrypanCruzi cruzi* discrete typing units in Chagas disease patients from endemic and non-endemic regions of Argentina. Parasitology. 2012;139:516–21. 22309735 10.1017/S0031182011002186

[88] Perez E, Monje M, Chang B, Buitrago R, Parrado R, Barnabé C, et al. Predominance of hybrid discrete typing units of *Trypanosoma Cruzi* in domestic *Triatoma infestans* from the Bolivian Gran Chaco region. Infect Genet Evol. 2013;13:116–23. 23047136 10.1016/j.meegid.2012.09.014

[89] Nawy T. Single-cell sequencing. Nat Methods. 2014;11:18. 24524131 10.1038/nmeth.2771

[90] Maestri S, Maturo MG, Cosentino E, Marcolungo L, Iadarola B, Fortunati E et al. A Long-Read sequencing Approach for Direct Haplotype phasing in clinical settings. Int J Mol Sci. 2020;21. 10.3390/ijms21239177 PMC7731377 33271988

[91] Kronenberg ZN, Rhie A, Koren S, Concepcion GT, Peluso P, Munson KM, et al. Extended haplotype-phasing of long-read de novo genome assemblies using Hi-C. Nat Commun. 2021;12:1935. 33911078 10.1038/s41467-020-20536-y PMC8081726

[92] Hosch S, Wagner P, Giger JN, Dubach N, Saavedra E, Perno CF, et al. PHARE: a bioinformatics pipeline for compositional profiling of multiclonal *Plasmodium falciparum* infections from long-read Nanopore sequencing data. J Antimicrob Chemother. 2024;79:987–96. 38502783 10.1093/jac/dkae060 PMC11062946

