# Supplementary material for: Detecting complex infections in trypanosomatids using whole genome sequencing
Source: BMC Genomics. 2024 Oct 29;25:1011. doi: 10.1186/s12864-024-10862-6 (PMC11520695; doi:10.1186/s12864-024-10862-6)
Supplement: Supplementary file 9 — Supplementary Material 9: Contains all the 16 supplementary figures. [file 12864_2024_10862_MOESM9_ESM.docx]

**Additional File 9 Supplementary_Figures_Review1:** Contains all the 16 supplementary figures


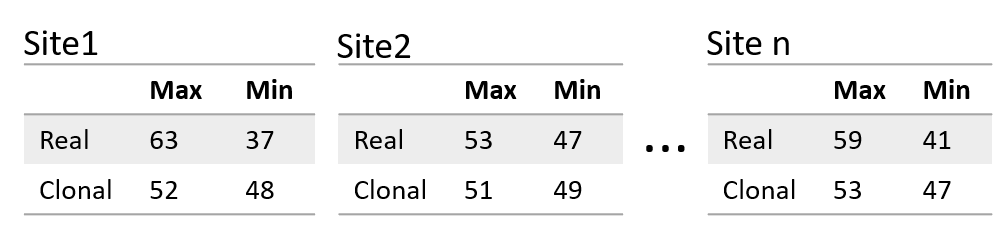


**Supplementary_Figure1: Contingency tables for the CMH test using heterozygous SNP data.** Each box represents a different SNP position from Site 1 to Site *n*, and the numbers represent the read depth in each allele in the position. Real: Data from real isolate. Clonal: Data from the simulated clonal isolate. Max: Allele with the largest read depth in that position; Min: Allele with the lowest read depth in the position.

**
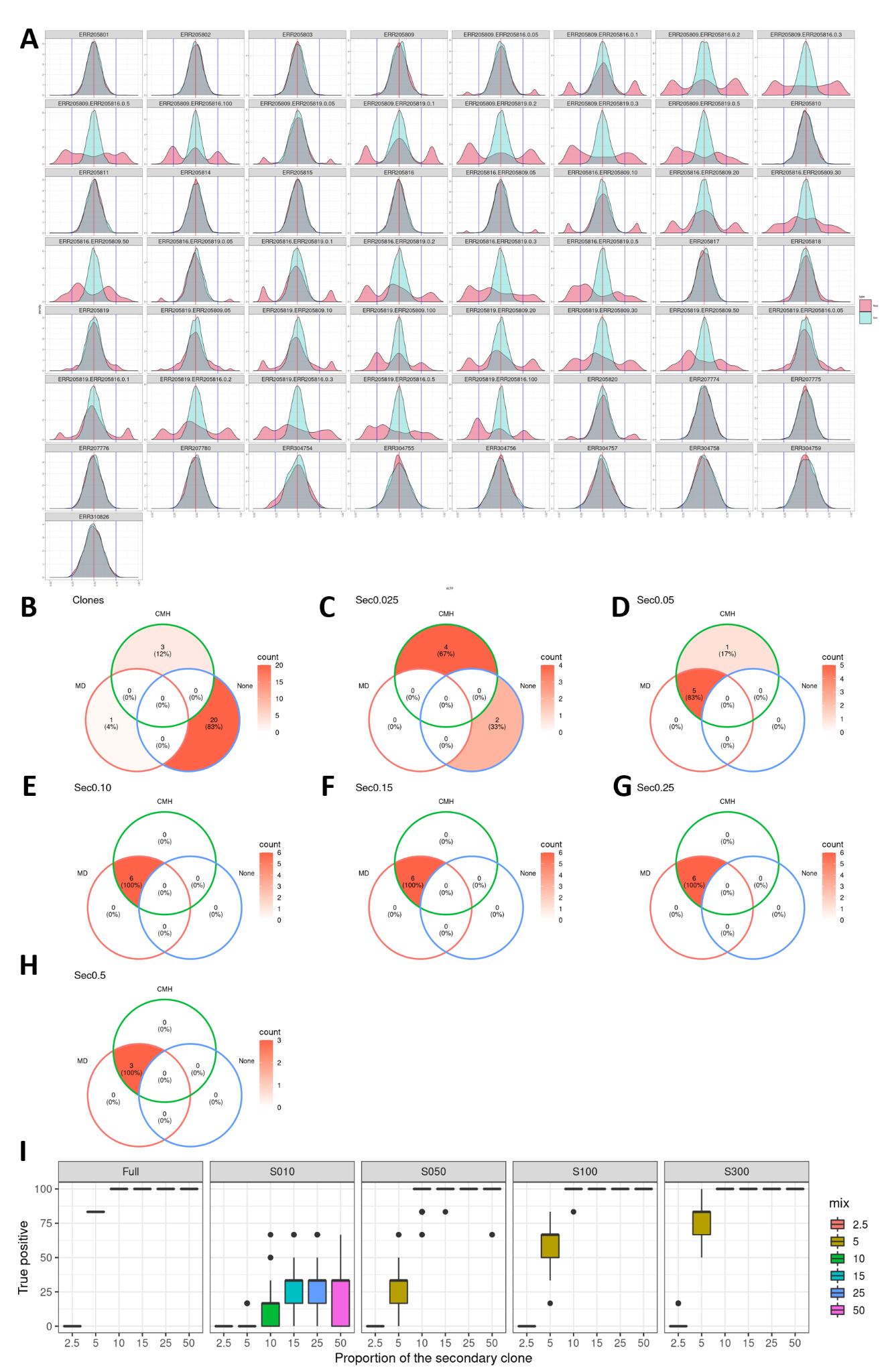
**

**Supplementary figure 2: Assessing the impact of the proportion if the secondary clone and heterozygous SNP counts in the CI estimations.** **A)** Density distributions of the AARD proportion in heterozygous positions for all combinations of the secondary clone, varying from 2.5-50%, as well as for the diploid clones. Each panel corresponds to a different isolate. The red distribution corresponds to the isolate data, while the cyan distribution represents the simulated clone, with the same number of SNPs and read depth as the real sample. **B - I)** Venn diagrams assessing the MD and CMH test accuracy to classify 24 clonal isolates (Clones), and 33 mixed infections (mix). Results that were below the cutoff for both are in the “None” quadrant. Each panel corresponds to the complexity estimation with a different proportion of the secondary clone, **B)** Clones; **C)** 2.5%; **D)** 5%; **E)** 10%; **F)** 15%; **G)** 25% and **H)** 50%. **I)** Impact of the number of SNPs in complexity estimations. Boxplot representing the percentage of MIX samples that were classified as complex. Each panel corresponds to downsampling to a different SNP count, where “S010”, “S050”, “S100”, “S300” and “Full” correspond, respectively, to 10, 50, 100, 300 and the Full SNP dataset. The X axis corresponds to the proportion of the secondary clone, from 2.5%-50% and the Y axis to the proportion of samples from each dataset that were correctly estimated as complex, in 100 iterations.


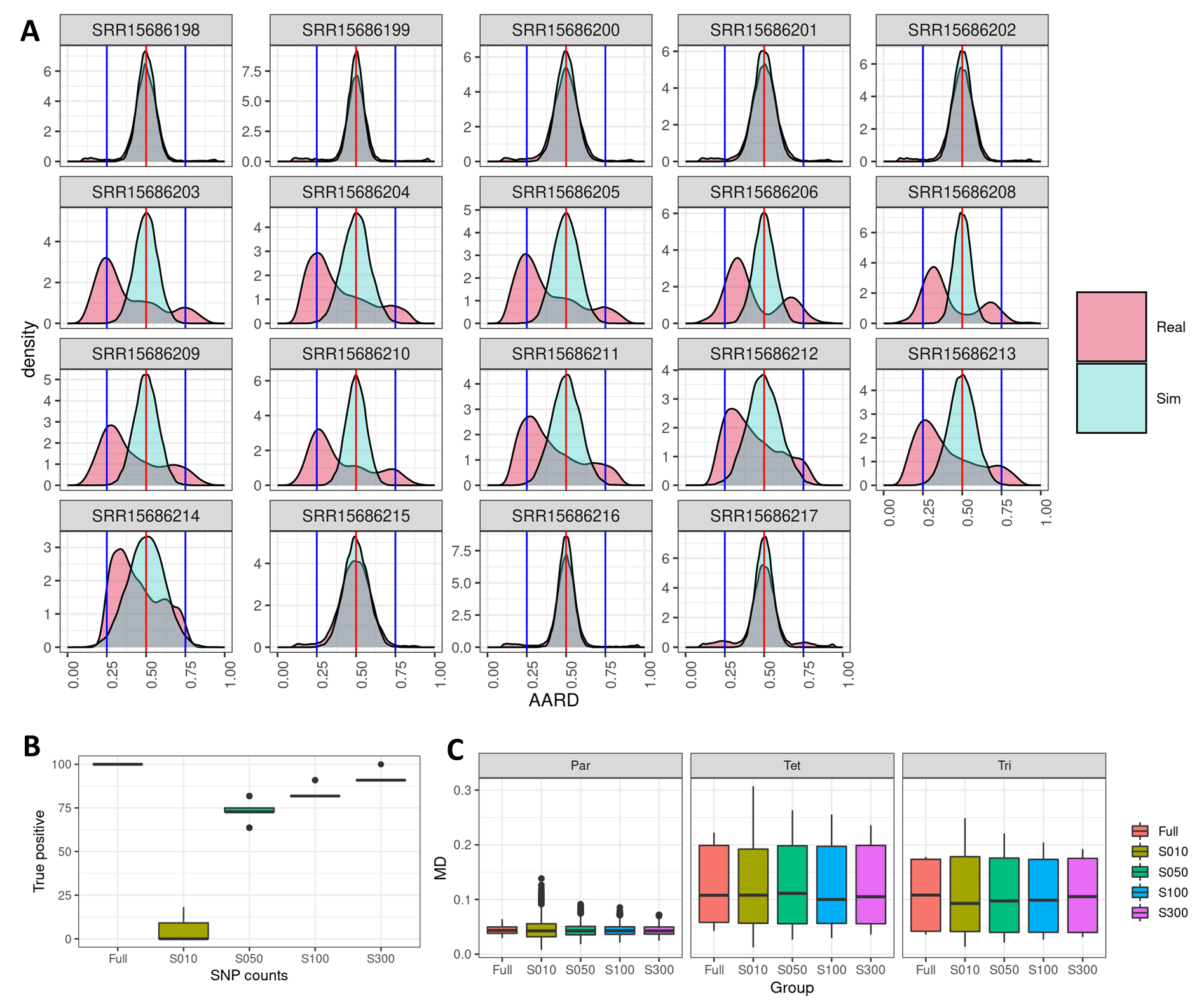


**Supplementary_Figure 3: The impact of ploidy variation in complexity. A)** Density distributions of the AARD proportion in heterozygous positions for all triploid ("SRR15686206","SRR15686208") and tetraploid ("SRR15686203", "SRR15686204", "SRR15686205", "SRR15686207", "SRR15686209", "SRR15686210", "SRR15686211", "SRR15686212", "SRR15686213", "SRR15686213", "SRR15686214") samples, as well as for the dissomic clones (“SRR15686198”, “SRR15686199”, “SRR15686200”, “SRR15686201”, “SRR15686202”, “SRR15686215”, “SRR15686216”, “SRR15686217”). The red distribution corresponds to the isolate data, while the cyan distribution represents the simulated clone, with the same number of SNPs and read depth as the real sample. **B)** Boxplots representing the impact of SNP counts in the accuracy of classifying polyploid isolates as complex, using 100 replicate interactions. **C)** Boxplots representing the impact of SNP counts in the complexity values. Each panel corresponds to a group: Parental Diploid (Par), Tetraploid (Tet) and Triploid (Tri) samples.


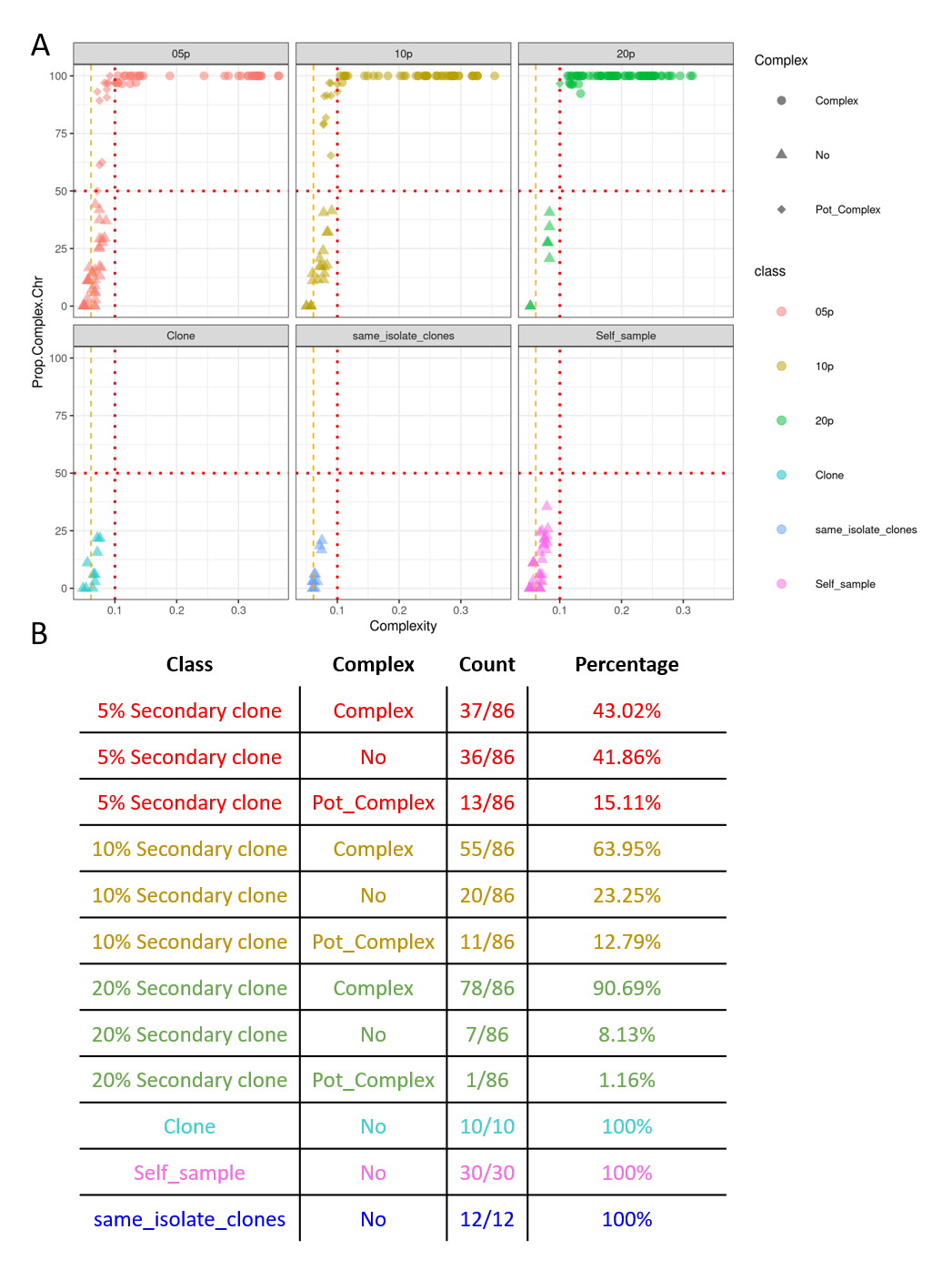


**Supplementary figure 4: Evaluating the complexity infection accuracy using 10 *T. cruzi* clones. A)** Complexity estimations in each sample. Each dot corresponds to a complex (circles), potential complex (diamond) or non-complex (triangles) isolates. The X and Y axis represents, respectively, the CI and proportion of the evaluated chromosomes that had a CI ≥ 0.1. Each group of samples is represented by a different panel. The colour corresponds to the sample origin, where 05p (red), 10p (yellow) and 20p (green) corresponds to the results where the secondary clone was respectively 5%, 10% and 20% of the sample. The “clones” panel corresponds to the clones of origin, and is represented by light blue. The “same isolates clone” correspond to the *in silico* mix of clones from the same primary isolate (but that were cultivated separately), and are represented in dark blue. Finally, the “self sample” panel corresponds to the *in silico* mix of a given clone to itself as is represented in pink. The orange vertical dotted lines represent complexity cutoff estimated based on the population data, while the red vertical line is the global complexity cutoff of 0.1, which separates the potential complex from the complex isolates. B) Table summarising the results for each group of samples.

**
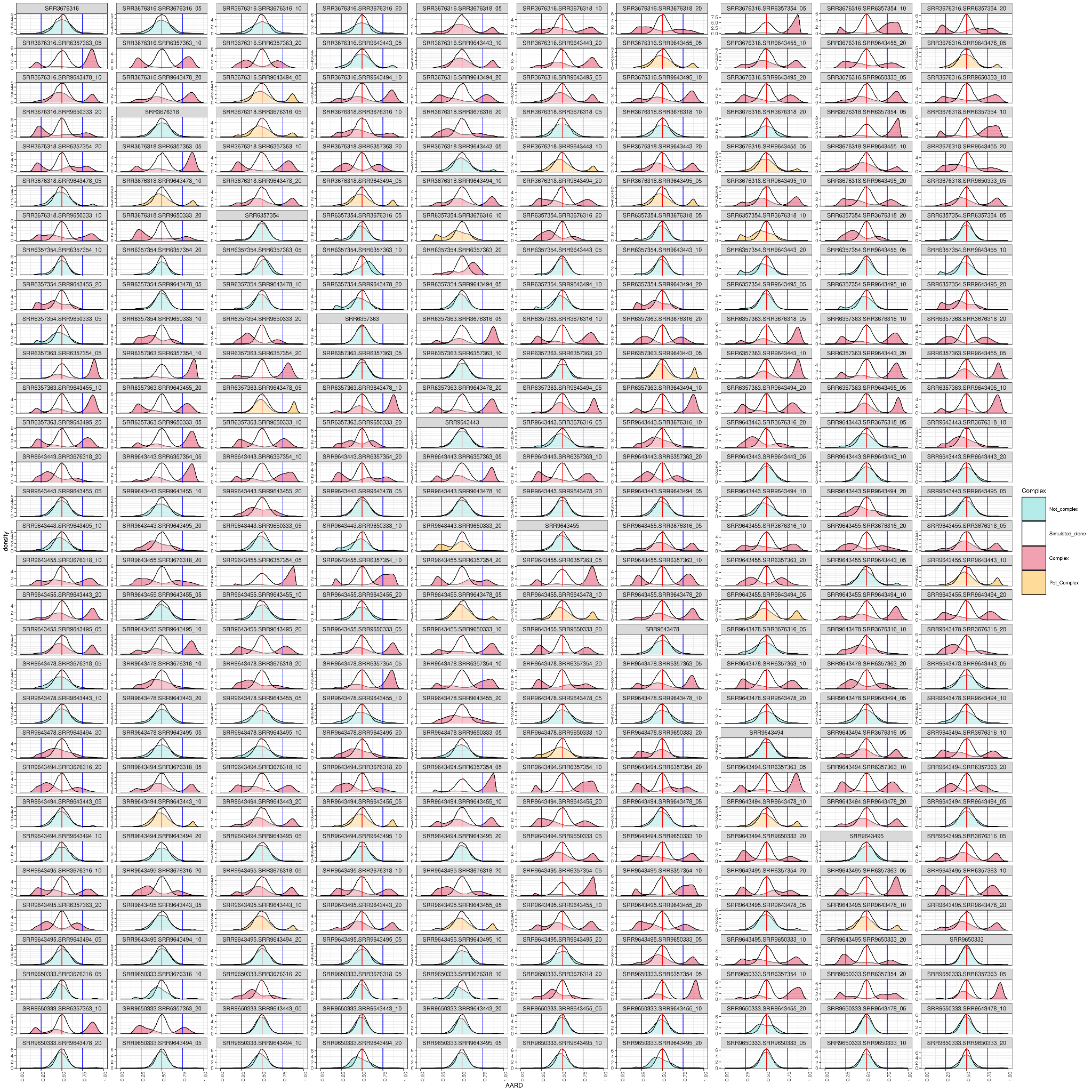
**

**Supplementary figure 5: Density distributions of the AARD proportion in heterozygous positions for all *T. cruzi* clones and mixed samples evaluated in the complexity pipeline evaluation stage**. The colour corresponds to the sample classification: complex (red), potential complex (orange) and non-complex (blue) isolates.


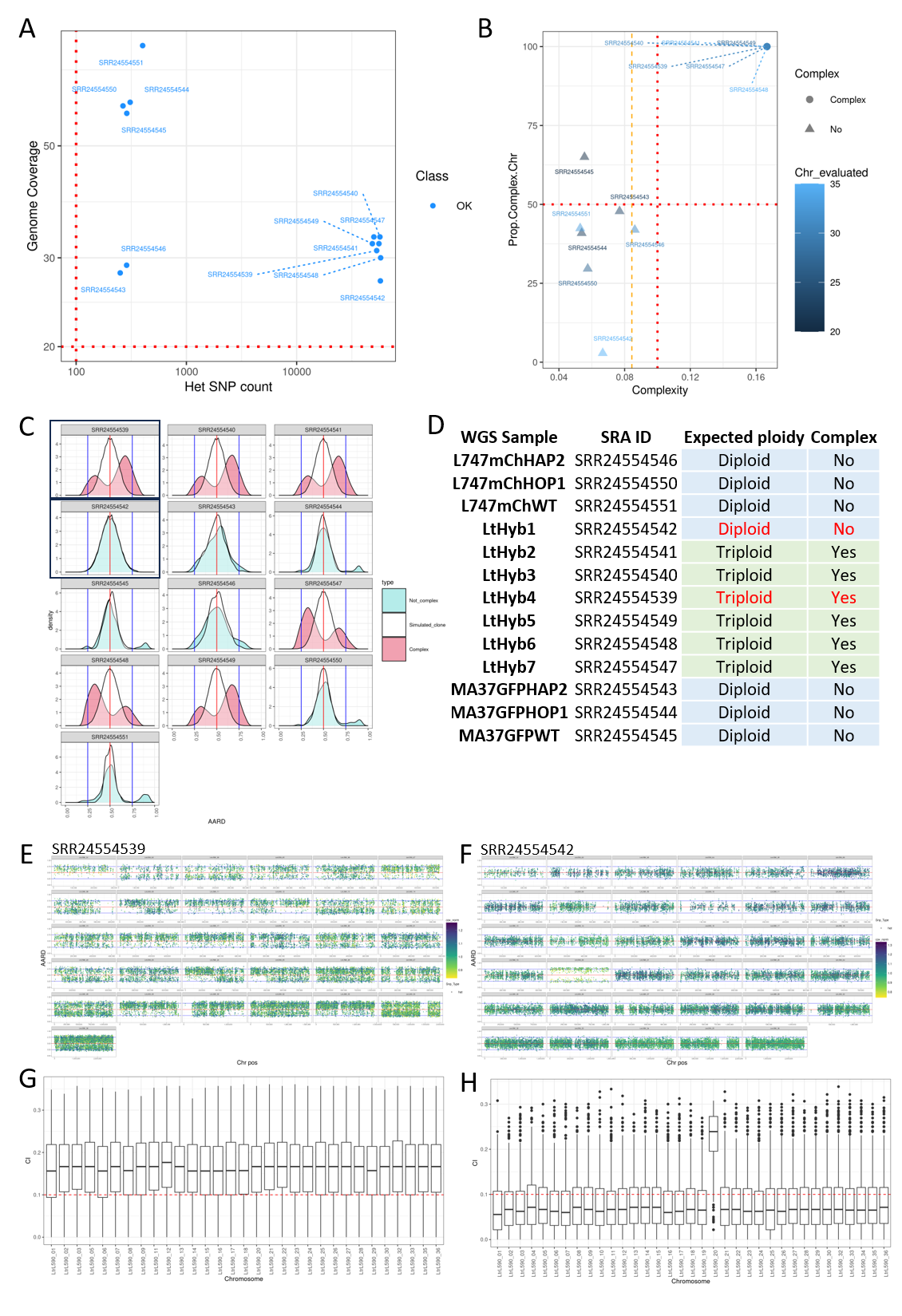


**Supplementary figure 6: Evaluation of the complexity of the *Leishmania tropica* parentals and hybrid samples from Cata Preta 2023**. A) Evaluation of the genome coverage and SNP counts of the samples. Samples with more than 25x coverage and 100 heterozygous SNPs are coloured in blue, while samples below any of these cutoffs are in red. **B)** Complexity estimations in each sample. Each dot corresponds to a complex (circles), potential complex (diamond) or non-complex (triangles) isolates. The X and Y axis represents, respectively, the CI and proportion of the evaluated chromosomes that had a CI ≥ 0.1. The colour corresponds to the proportion of chromosomes that were evaluated in the isolate. The orange vertical dotted lines represent complexity cutoff estimated based on the population data, while the red vertical line is the global complexity cutoff of 0.1, which separates the potential complex from the complex isolates. **C)** AARD distribution from the complex (red), potential complex (orange) and non-complex (blue) isolates. **D)** Table summarising the sample SRA IDs and names, the “expected ploidy” based on Cata Preta’s descriptions and “complex” our classification of complexity. Samples in red highlight examples used in E-H. **E)** and **F)** correspond respectively to AARD value (y axis) for each SNP in each chromosome (pannel) position (x axis), from a complex (SRR24554539) and a non-complex (SRR24554542) sample. **G)** and **H)** correspond respectively to boxplots of the complexity of SNPs in each chromosome from a complex (SRR24554539) and a non-complex (SRR24554542) sample.


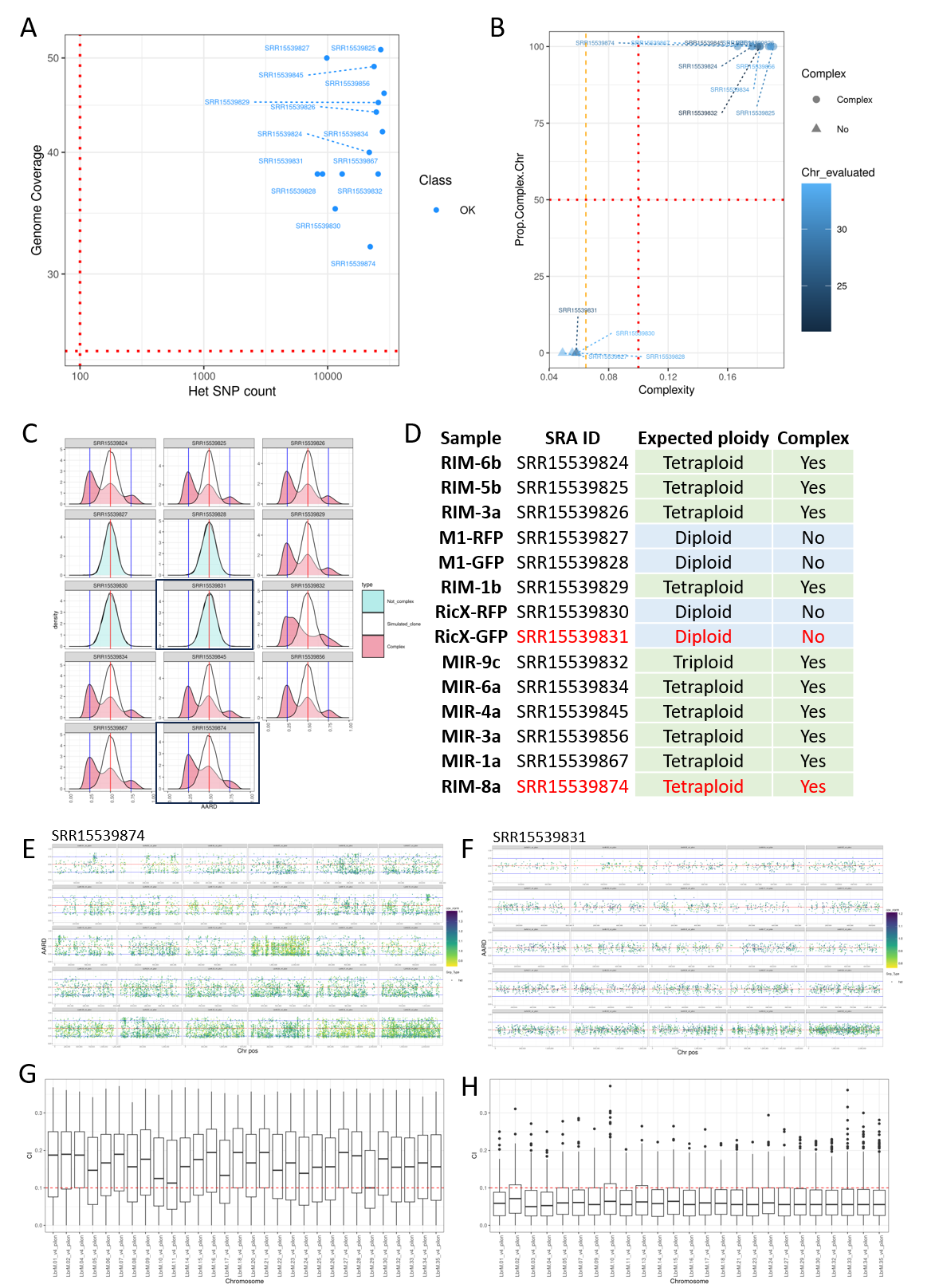


**Supplementary figure 7: Evaluation of the complexity of the *Leishmania braziliensis* parentals and hybrid samples from Lourador 2022**. **A)** Evaluation of the genome coverage and SNP counts of the samples. Samples with more than 25x coverage and 100 heterozygous SNPs are coloured in blue, while samples below any of these cutoffs are in red. **B)** Complexity estimations in each sample. Each dot corresponds to a complex (circles), potential complex (diamond) or non-complex (triangles) isolates. The X and Y axis represents, respectively, the CI and proportion of the evaluated chromosomes that had a CI ≥ 0.1. The colour corresponds to the proportion of chromosomes that were evaluated in the isolate. The orange vertical dotted lines represent complexity cutoff estimated based on the population data, while the red vertical line is the global complexity cutoff of 0.1, which separates the potential complex from the complex isolates. **C)** AARD distribution from the complex (red), potential complex (orange) and non-complex (blue) isolates. **D)** Table summarising the sample SRA IDs and names, the “expected ploidy” based on Lorador’s descriptions and “complex” our classification of complexity. Samples in red highlight examples used in E-H. **E)** and **F)** correspond respectively to AARD value (y axis) for each SNP in each chromosome (pannel) position (x axis), from a complex (SRR15539874) and a non-complex (SRR15539874) sample. **G)** and **H)** correspond respectively to boxplots of the complexity of SNPs in each chromosome from a complex (SRR15539874) and a non-complex (SRR15539831) sample.


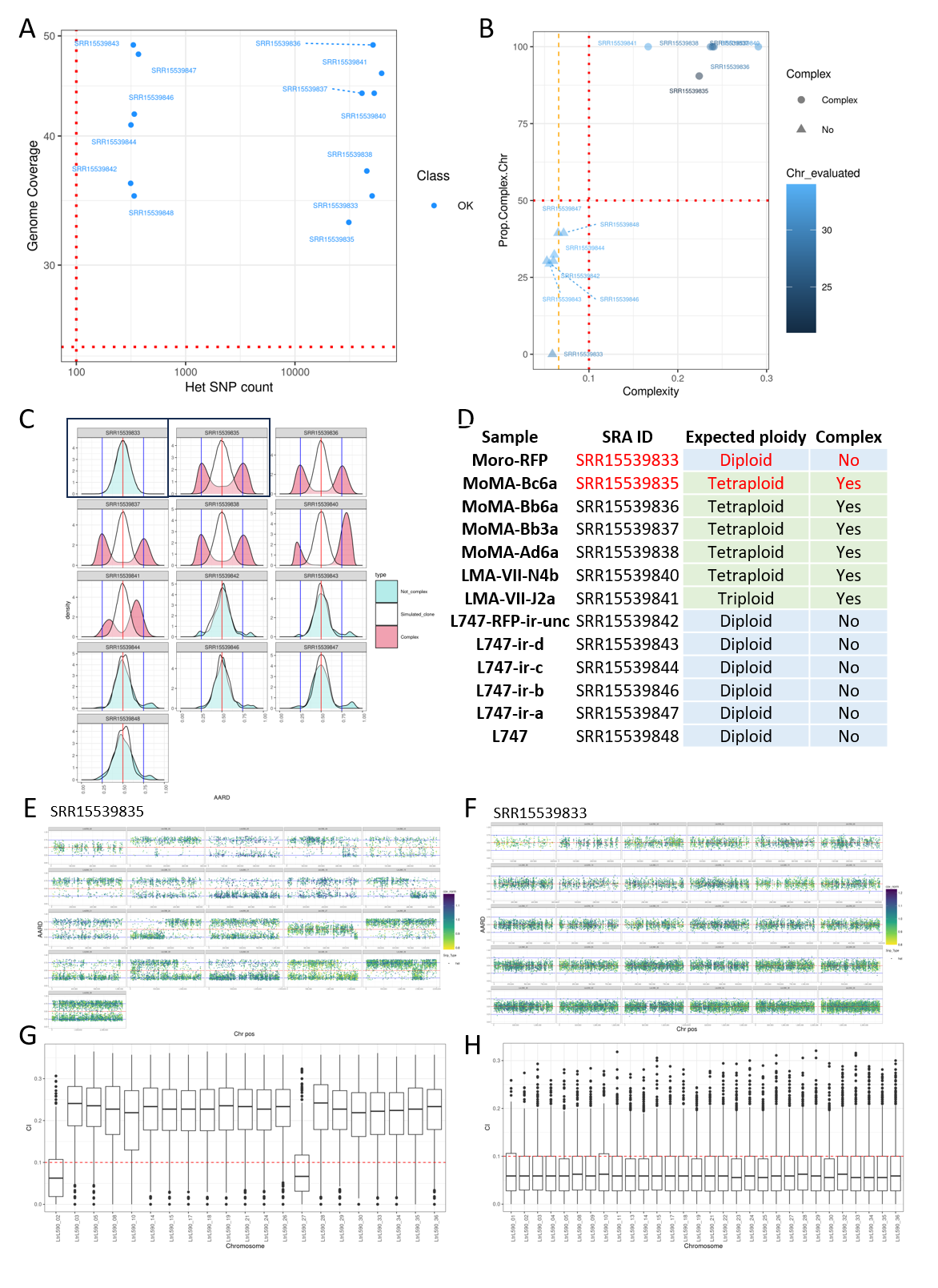


**Supplementary figure 8: Evaluation of the complexity of the *Leishmania tropica* parentals and hybrid samples from Lourador 2022**. **A)** Evaluation of the genome coverage and SNP counts of the samples. Samples with more than 25x coverage and 100 heterozygous SNPs are coloured in blue, while samples below any of these cutoffs are in red. **B)** Complexity estimations in each sample. Each dot corresponds to a complex (circles), potential complex (diamond) or non-complex (triangles) isolates. The X and Y axis represents, respectively, the CI and proportion of the evaluated chromosomes that had a CI ≥ 0.1. The colour corresponds to the proportion of chromosomes that were evaluated in the isolate. The orange vertical dotted lines represent complexity cutoff estimated based on the population data, while the red vertical line is the global complexity cutoff of 0.1, which separates the potential complex from the complex isolates. **C)** AARD distribution from the complex (red), potential complex (orange) and non-complex (blue) isolates. **D)** Table summarising the sample SRA IDs and names, the “expected ploidy” based on Lorador’s descriptions and “complex” our classification of complexity. Samples in red highlight examples used in E-H. **E)** and **F)** correspond respectively to AARD value (y axis) for each SNP in each chromosome (pannel) position (x axis), from a complex (SRR15539835) and a non-complex (SRR15539833) sample. **G)** and **H)** correspond respectively to boxplots of the complexity of SNPs in each chromosome from a complex (SRR15539835) and a non-complex (SRR15539833) sample.


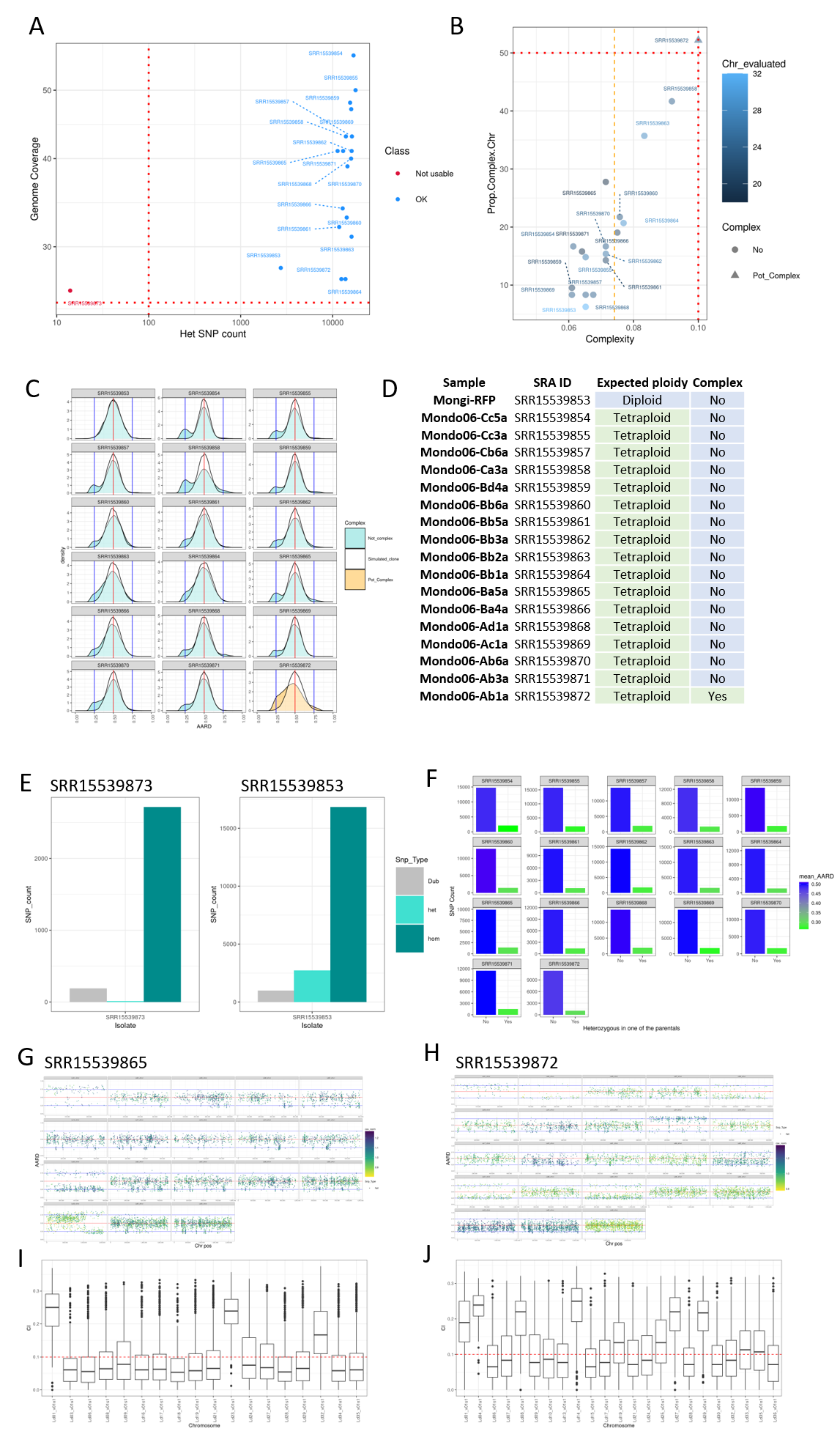


**Supplementary figure 9: Evaluation of the complexity of the *Leishmania donovani* parentals and hybrid samples from Lourador 2022**. **A)** Evaluation of the genome coverage and SNP counts of the samples. Samples with more than 25x coverage and 100 heterozygous SNPs are coloured in blue, while samples below any of these cutoffs are in red. **B)** Complexity estimations in each sample. Each dot corresponds to a complex (circles), potential complex (diamond) or non-complex (triangles) isolates. The X and Y axis represents, respectively, the CI and proportion of the evaluated chromosomes that had a CI ≥ 0.1. The colour corresponds to the proportion of chromosomes that were evaluated in the isolate. The orange vertical dotted lines represent complexity cutoff estimated based on the population data, while the red vertical line is the global complexity cutoff of 0.1, which separates the potential complex from the complex isolates. **C)** AARD distribution from the complex (red), potential complex (orange) and non-complex (blue) isolates. **D)** Table summarising the sample SRA IDs and names, the “expected ploidy” based on Lorador’s descriptions and “complex” our classification of complexity. Samples in red highlight examples used in G-J. **E)** Heterozygous (light-green), Homozygous (green) and Dubious (grey - SNPs that had more than 1 but less than 5 reads in the minor allele). **F)** Evaluation of the heterozygous SNPs in the hybrid strains. The column “No” corresponds to SNPs in the hybrid strains that were not heterozygous exclusive to one parental strain (i.e was homozygous in one parental strain or heterozygous in both). The column “Yes” corresponds to the heterozygous SNPs in the hybrid strains that were heterozygous in only one of the two parental strains. The bars are coloured based on AARD values, where blue corresponds to ~0.5 and green ~0.25. **G)** and **H)** correspond respectively to AARD value (y axis) for each SNP in each chromosome (pannel) position (x axis), from SRR15539865 and SRR15539872 samples. **I)** and **J)** correspond respectively to boxplots of the complexity of SNPs in each chromosome from SRR15539865 and SRR15539872.


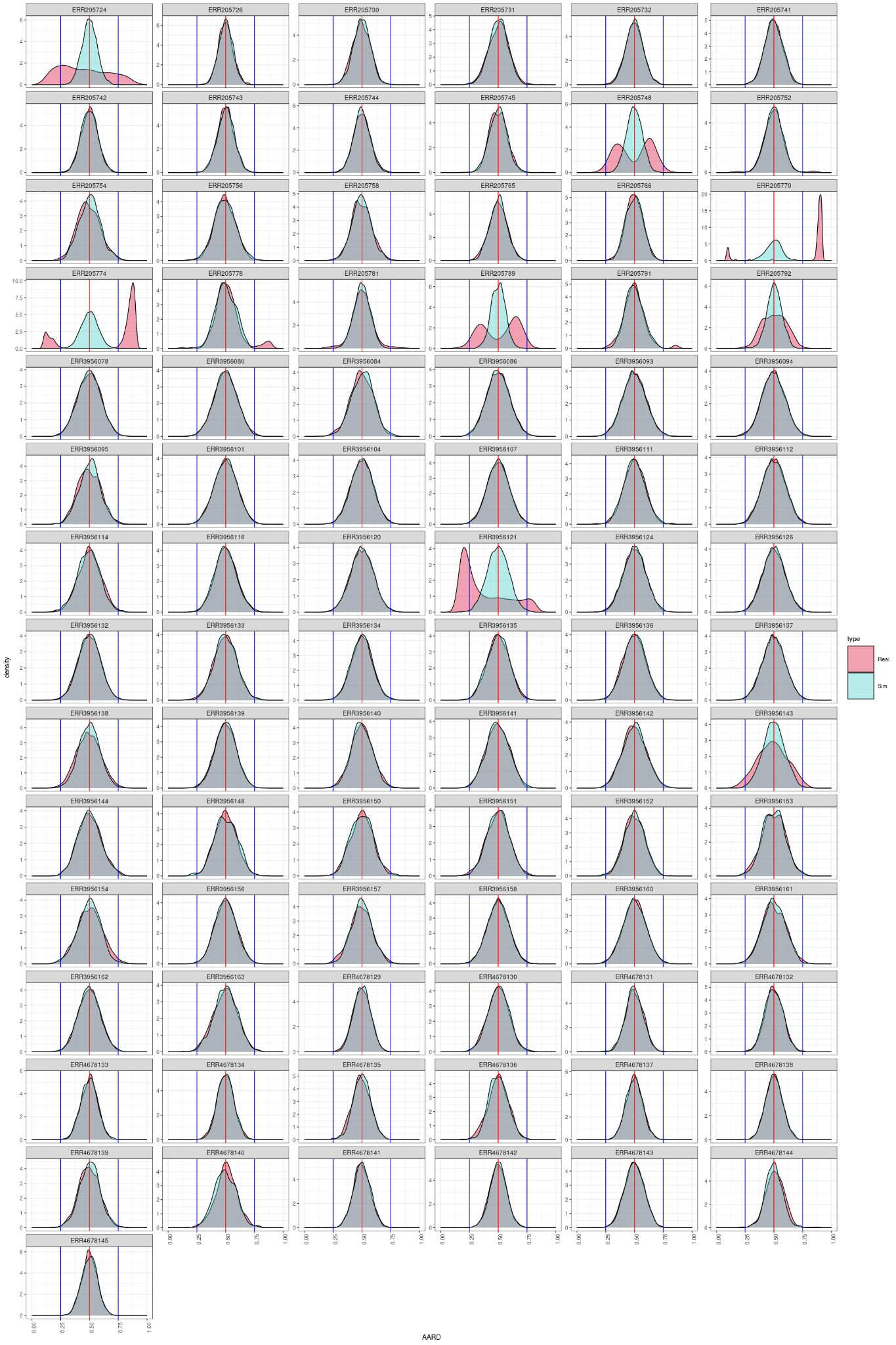


**Supplementary figure 10: AARD density plot for the 85 *L. donovani* samples.**

**
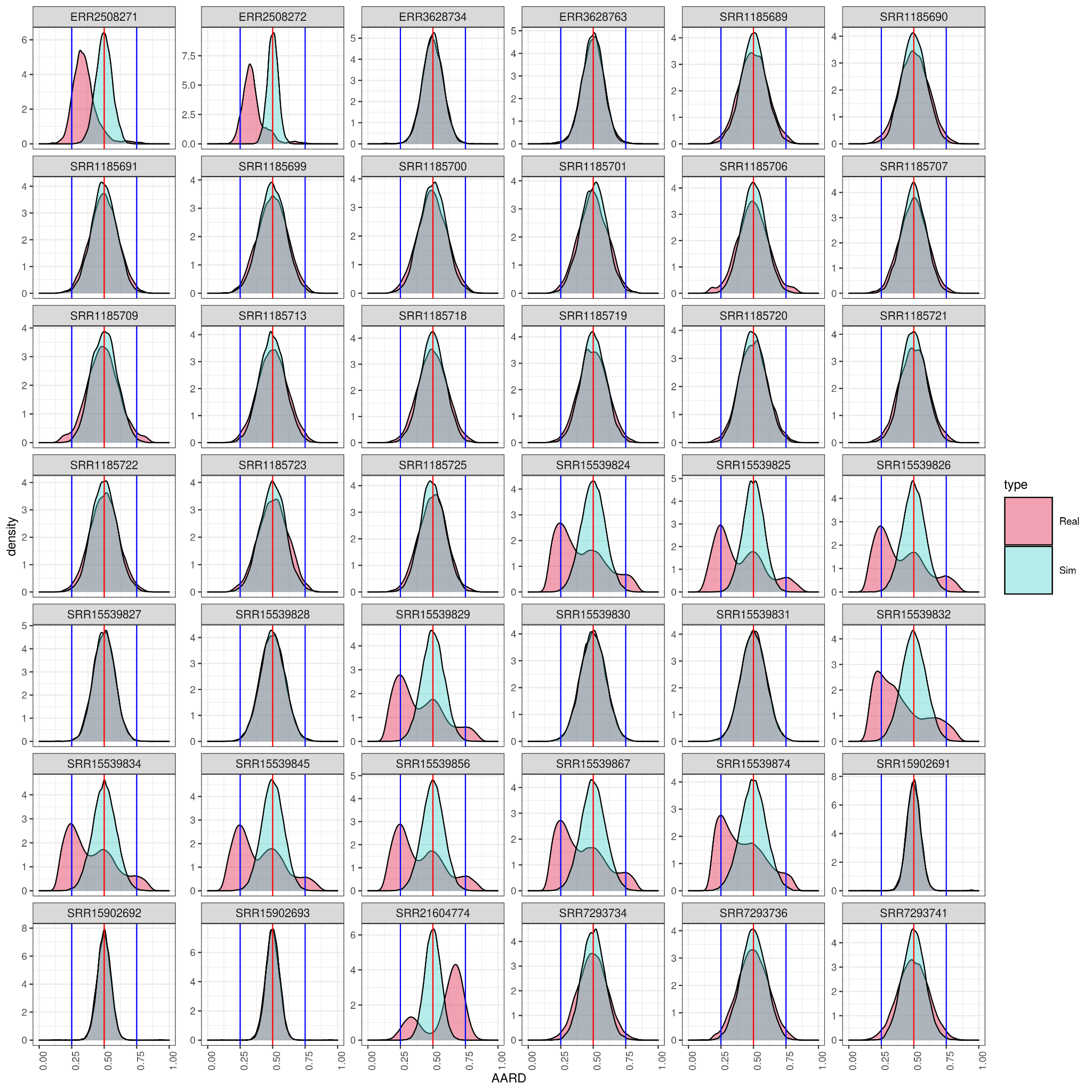
**

**Supplementary figure 11: AARD density plot for the 42 *L. braziliensis* samples.**

**
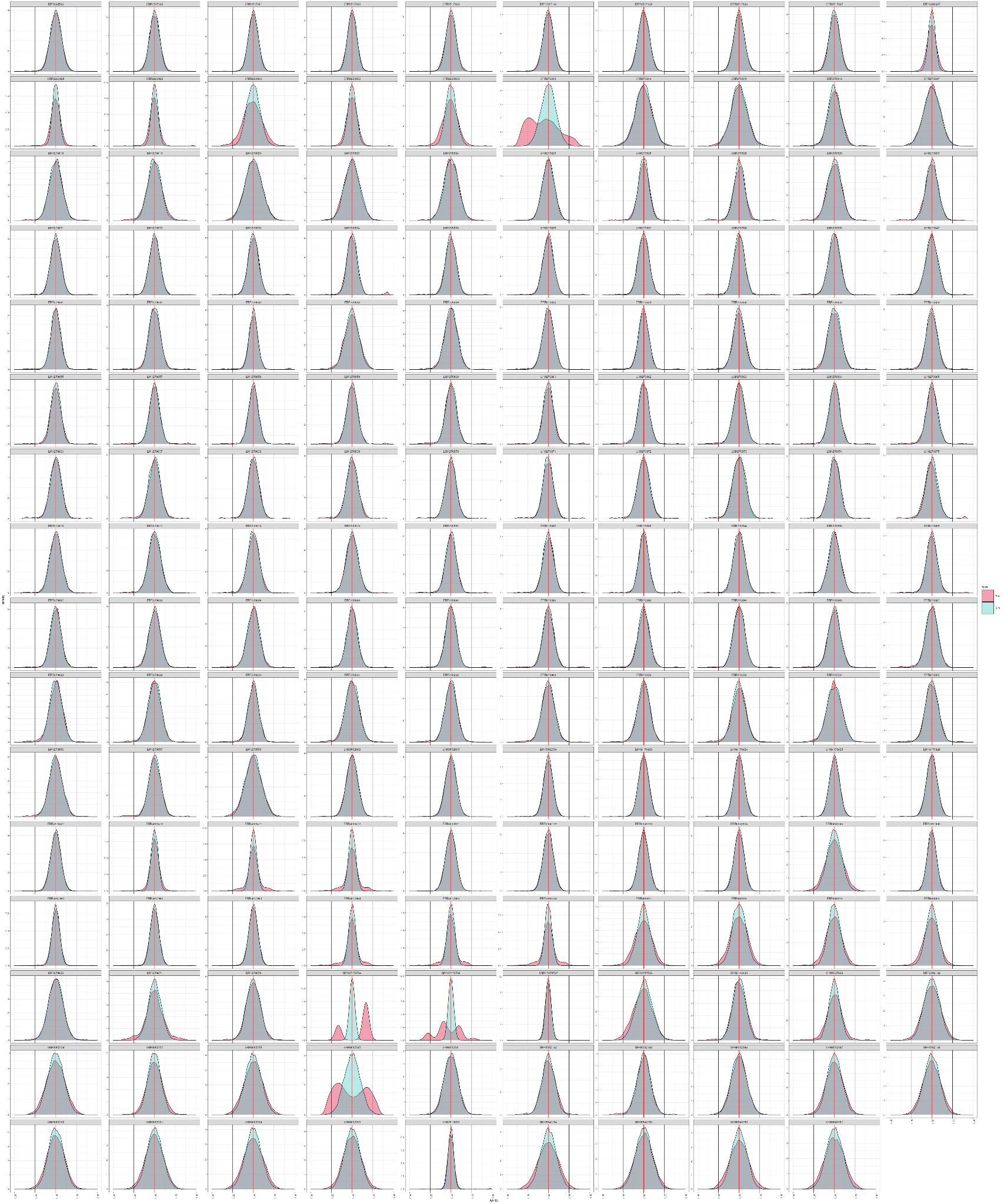
**

**Supplementary figure 12: AARD density plot for the 159 *T. brucei* samples.**

**
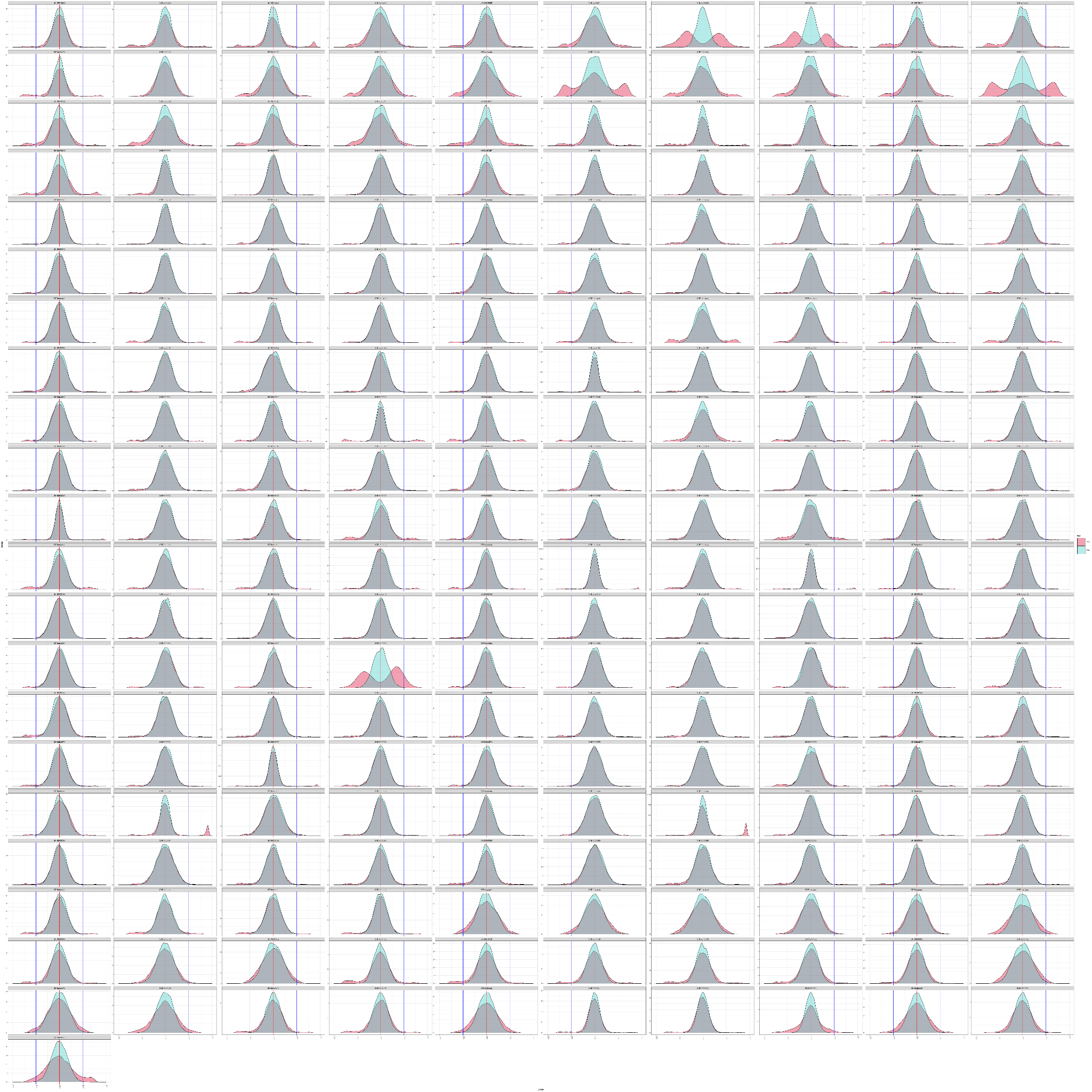
**

**Supplementary figure 13: AARD density plot for the 211*T. cruzi* samples.**


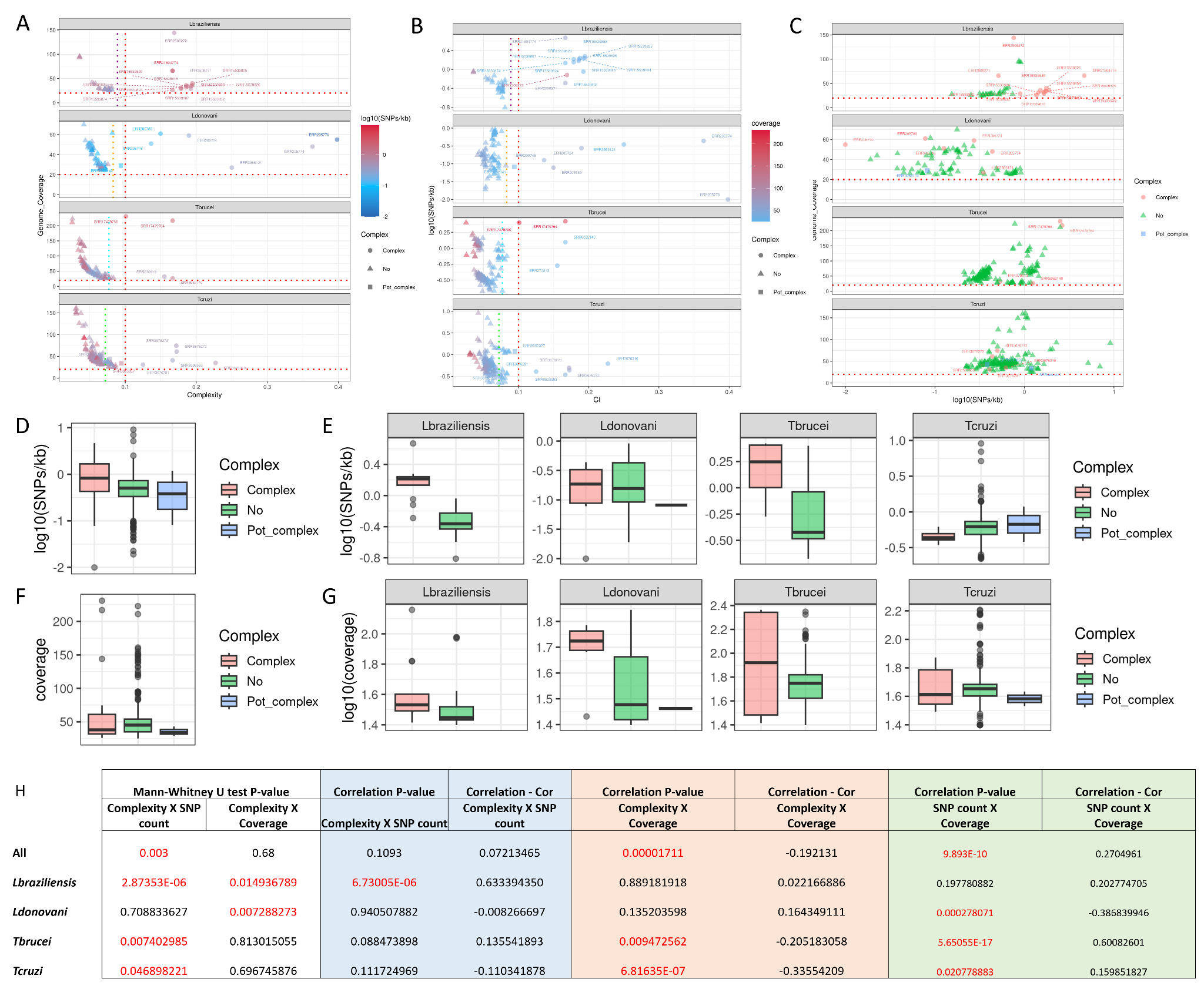


**Supplementary figure 14: Evaluation of genome coverage, SNP count on complexity estimations in field and lab trypanosomatid isolates. A-C)** Genome coverage and mean complexity in each sample; **B)** log10(SNPs/kb) counts and mean complexity in each sample; **C)** Genome coverage and log10(SNPs/kb) in each sample. In these plots, each panel corresponds to a different species: from top to bottom *L. braziliensis*, *L.donovani*, *T.brucei* and *T.cruzi*, while each dot corresponds to a different isolate. The vertical red line in A and C corresponds to the 0.1 complexity cutoff, while the coloured lines correspond to the species population complexity cutoff. The horizontal red dotted lines in A and C correspond to the genome coverage value of 20. Boxplots representing the SNP counts, grouped by “Complex” (red), “Not complex” (green) and “Potential complex” (blue) for **D)** all samples; or **E)** separated by species. Boxplots representing the genome coverage for **F)** all samples; or **G)** separated by species. **H)** Table with the Mann-Whitney U test and Correlation between complexity, heterozygous SNP counts and genome coverage.


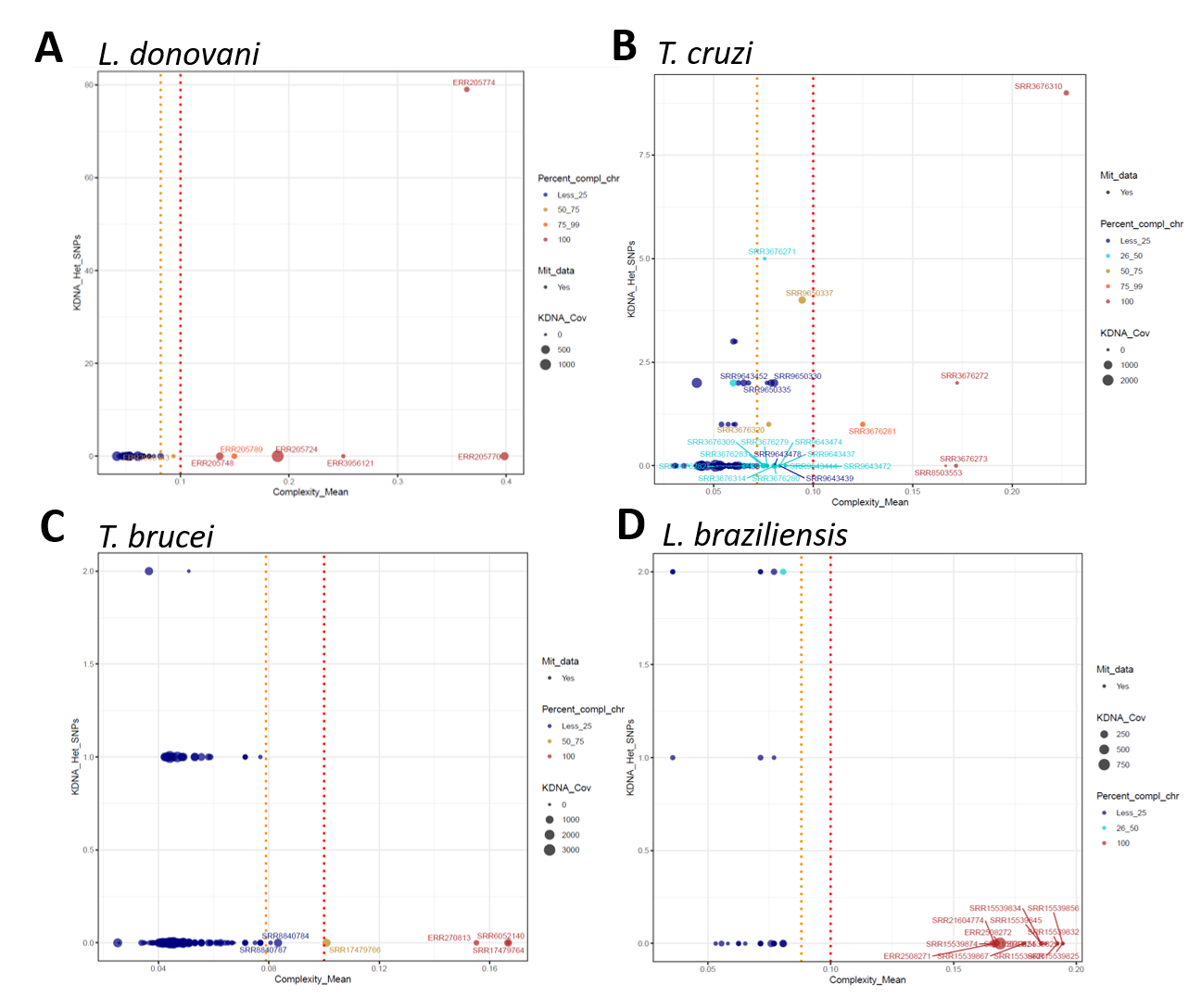


**Supplementary figure 15: Maxicircle DNA heterozygous SNPs and the complexity estimated for the nuclear genome.** In this figure, each dot corresponds to a parasite sample, the “Y” axis to the number of Heterozygous SNPs in the Maxicircle (mitochondrial) genome; and the “X” axis to the nuclear complexity of the isolate. The size of the dot represents the Maxicircle genome coverage, and the colours correspond to the proportion of nuclear chromosomes that had a mean complexity higher than 0.1.


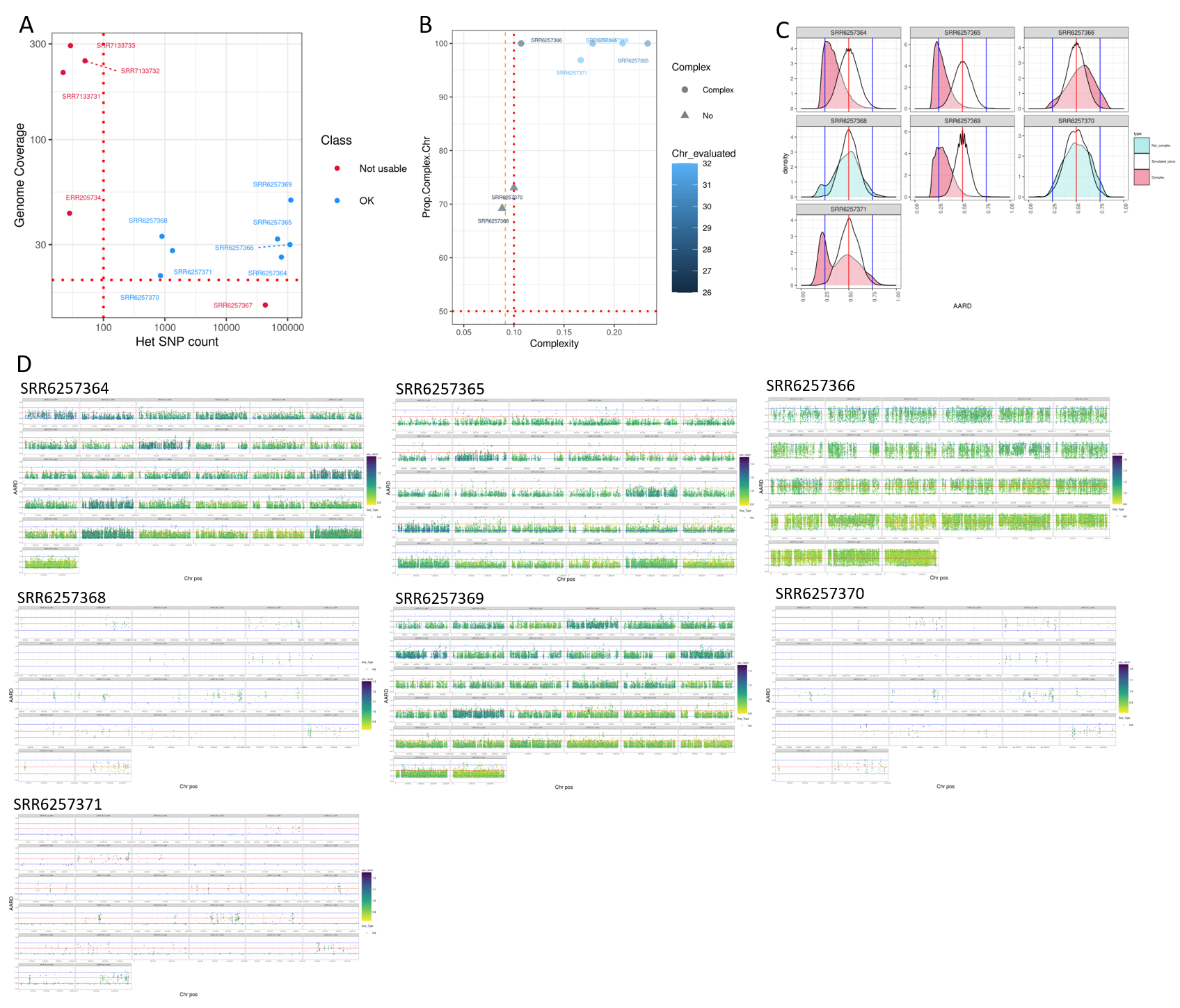


**Supplementary figure 16: Evaluation of the complexity of the Sri Lanka hybrid isolates from Lypaczewski 2021**. **A)** Evaluation of the genome coverage and SNP counts of the samples. Samples with more than 25x coverage and 100 heterozygous SNPs are coloured in blue, while samples below any of these cutoffs are in red. **B)** Complexity estimations in each sample. Each dot corresponds to a complex (circles), potential complex (diamond) or non-complex (triangles) isolates. The X and Y axis represents, respectively, the CI and proportion of the evaluated chromosomes that had a CI ≥ 0.1. The colour corresponds to the proportion of chromosomes that were evaluated in the isolate. The orange vertical dotted lines represent complexity cutoff estimated based on the population data, while the red vertical line is the global complexity cutoff of 0.1, which separates the potential complex from the complex isolates. **C)** AARD distribution from the complex (red), potential complex (orange) and non-complex (blue) isolates. **D)** AARD value (y axis) for each SNP in each chromosome (panel) position (x axis), for the evaluated Sri Lanka samples. SRR6257364, SRR6257365, SRR6257366 and SRR6257369 were classified as SL2, while SRR6257368, SRR6257370 and SRR6257371 were classified as SL3 by Lypaczewski 2021.
